# hnRNPL–CstF64 complex: coordinating CSR and LSR in IgH locus recombination dynamics through eRNA and NHEJ regulation

**DOI:** 10.1093/nar/gkaf810

**Published:** 2025-09-03

**Authors:** Farazul Haque, Mikiyo Nakata, Hidetaka Kosako, Tasuku Honjo, Nasim A Begum

**Affiliations:** Department of Immunology and Genomic Medicine, Centre for Cancer Immunotherapy and Immunobiology, Kyoto University Graduate School of Medicine, Kyoto 606-8501, Japan; Department of Immunology and Genomic Medicine, Centre for Cancer Immunotherapy and Immunobiology, Kyoto University Graduate School of Medicine, Kyoto 606-8501, Japan; Division of Cell Signaling, Institute of Advanced Medical Sciences, Tokushima University, Tokushima 770-8503, Japan; Department of Immunology and Genomic Medicine, Centre for Cancer Immunotherapy and Immunobiology, Kyoto University Graduate School of Medicine, Kyoto 606-8501, Japan; Department of Immunology and Genomic Medicine, Centre for Cancer Immunotherapy and Immunobiology, Kyoto University Graduate School of Medicine, Kyoto 606-8501, Japan

## Abstract

Class switch recombination (CSR) and locus suicide recombination (LSR) are critical processes involved in the immune system’s ability to diversify antibody responses. Both are initiated by activation-induced cytidine deaminase, which induces DNA double-strand breaks (DSBs) at specific regions within the immunoglobulin heavy chain (IgH) locus. In CSR, DSBs occur at the switch (S) regions, allowing B cells to replace the IgM heavy chain constant region (CH) with other isotypes, thereby enhancing immune adaptability. This process is regulated by both *cis* and *trans* mechanisms, including the IgH super-enhancer 3′ regulatory region (3′RR) and the production of enhancer RNAs (eRNAs). A recent study highlighted the role of MED12 in CSR through enhancer activation and the transcription of eRNA. Now, we show that heterogeneous ribonucleoprotein L (hnRNPL) acts as an additional regulator of CSR and LSR by forming an eRNA-associated complex with CstF64, a polyadenylation factor. This complex facilitates RNA polymerase II elongation and eRNA transcription at the 3′RR. Moreover, the hnRNPL/CstF64 complex promotes NHEJ-mediated DNA repair at both S and 3′RR regions, facilitating 53BP1 and Ku80 recruitment, thereby impacting the efficiency of CSR and LSR. This discovery highlights the intricate, multimodal regulation of these processes, linking eRNA transcription to DNA repair in the process of antibody diversification.

## Graphical Abstract

**Graphical Abstract:**
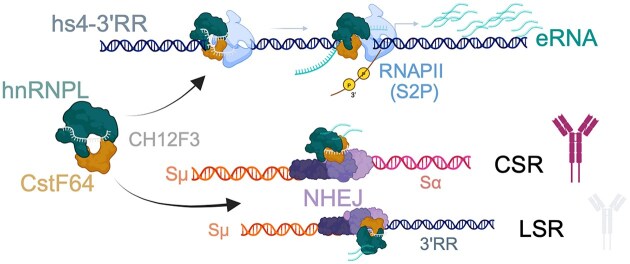

## Introduction

Adaptive immunity relies on B cells’ capacity to generate high-affinity, antigen-specific antibodies. Two critical DNA alteration mechanisms facilitate this process: somatic hypermutation (SHM) and class switch recombination (CSR), which are initiated by the expression of activation-induced cytidine deaminase (AID) [1, 2]. The SHM introduces extensive point mutations in the V exon of immunoglobulin genes, enhancing antibody affinity. In contrast, CSR involves the replacement of the immunoglobulin heavy chain (IgH) constant region gene, allowing B cells to switch from expressing IgM to other isotypes such as IgG, IgE, or IgA [3, 4]. CSR proceeds through two major steps: first, AID induces DNA double-strand breaks (DSBs) at actively transcribed switch (S) regions. Subsequently, these breaks are repaired by joining recombining donor and acceptor S regions within a recombination hub, often called the S–S synapse. The two-step process can be facilitated by multiple regulatory mechanisms, among which the regulation of locus-specific conformation plays a central role. This regulation delineates the topologically associated domains, influencing various processes, including S–S synapse formation, chromatin extrusion, and joining the two DSB ends through nonhomologous end joining (NHEJ) [5, 6].

The IgH super-enhancer, or 3′ regulatory region (3′RR), plays a crucial role in the basal and induced architecture of the IgH locus [7, 8]. It brings S regions closer together and enables their transcriptional activation, producing switch germline transcripts (GLTs) and noncoding enhancer RNAs (eRNAs) [9, 10]. These eRNAs influence histone modifications within the IgH locus, AID recruitment, and possibly other functions yet to be explored. The 3′RR spans ∼28 kb and contains four B-cell-specific enhancers: hs3a, hs1.2, hs3b, and hs4, which work together to regulate intra-IgH interactions and CSR efficiency [11, 12]. Recent studies have highlighted the role of factors such as SPT5 and ZMYND8 in regulating 3′RR activity, affecting RNA Polymerase II (RNAPII) loading and enhancer transcription [9, 13]. Enhancer transcription and activation are distinguished by the synthesis of the eRNAs, which are mostly short, bidirectionally transcribed, unspliced, and non-polyadenylated [14]. Despite the overwhelming evidence that eRNAs are functional biomolecules, the precise method by which they function remains unknown. However, various possible modes of action are emerging, including enhancer-promoter interactions, chromatin modifications, DNA repair, and transcriptional machinery modulation [15, 16]. Our recent findings demonstrate that Med12 is essential for transcribing 3′RR eRNAs, influencing the histone epigenetics of the S region and AID-induced DSBs, thereby impacting the constitutive and inducible S–S synaptic conformation of the IgH locus [10].

There are likely more factors governing the 3′RR activation and eRNA regulation in the dynamics of IgH locus recombination, which await further exploration. Several heterogeneous ribonucleoprotein (hnRNP) family proteins were found to be required for CSR, influencing either S region DSB or S–S recombination [17, 18]. Multiple members of the hnRNP family interact with AID, including hnRNPL, which was postulated as a DNA-repair-specific factor for CSR [18]. However, the precise mechanism by which hnRNPL regulates CSR and participates in DNA repair remains unclear. We unexpectedly revealed that hnRNPL acts as a novel transcriptional modulator of the eRNAs synthesized from the 3′RR. It also binds to eRNAs through its unique variant RNA recognition motif (vRRM) region and interacts with CstF64, a component of the polyadenylation machinery. The complex promotes RNAPII phosphorylation and eRNA transcription at the IgH 3′RR.

Notably, the hnRNPL-CTF64 complex also plays a role in repairing DSBs through NHEJ by stabilizing the NHEJ repair factors, impacting both CSR and locus suicide recombination (LSR), an uncommon rearrangement occurring between the S region and 3′RR DSBs [19, 20]. In LSR, AID induces DSBs at the 3′RR, leading to recombination between Sμ region and the 3′RR (Sμ-3′RR), resulting in the deletion of the intervening constant (C) gene cluster and the permanent inactivation of the IgH locus. While related to CSR and requiring NHEJ, this process has a negative consequence: it prevents further immunoglobulin production and may trigger programmed cell death in B cells with faulty recombination. This mechanism ensures that cells with aberrant recombination are eliminated, which may influence B cell affinity maturation and survival [21]. The hnRNPL–eRNA–CSTF64 complex thus reveals a novel regulatory axis coordinating CSR and LSR, highlighting its role in antibody diversification and protecting against defective recombination events.

## Materials and methods

### CH12F3-2A cells and CSR assay

A derivative of the CH12F3 mouse B cell lymphoma line, referred to as CH12F3-2A, was used throughout the study [22]. To improve survival following siRNA-mediated gene knockdown, we used a stable *Bcl2*-expressing CH12F3-2A line [23]. Cells were cultured in RPMI 1640 medium supplemented with 10% fetal bovine serum, l-glutamine, NCTC-109, 50 μM β-mercaptoethanol, and penicillin/streptomycin. CSR to IgA was induced by stimulation with CIT, a cytokine cocktail containing CD40L, IL-4, and TGF-β [22]. For surface staining, cells were labeled with FITC-conjugated anti-mouse IgM and PE-conjugated anti-mouse IgA antibodies, and dead cells were excluded using propidium iodide (PI). Flow cytometry was performed on a BD FACSCalibur, and data were analyzed using BD CellQuest software, with occasional use of FlowJo.

### Generation of *Cstf2* knockout CH12F3-2A cells

Guide RNAs (gRNAs) targeting *Cstf2* were designed using the CRISPOR online tool. The annealed gRNAs were cloned into the Guide-it™ CRISPR/Cas9 vector (Takara Bio, 632601) following the manufacturer’s instructions. Transfected cells were subjected to single-cell dilution to isolate individual clones. Genomic DNA (gDNA) from these clones was screened by PCR for gene editing, and the loss of CstF2 protein in hetro/homozygous knockout (KO) was confirmed via western blotting (Supplementary Fig. S2A–C). Several KO clones were also verified for the integrity of AID expression. The sequences of the gRNAs used for targeting are listed in Supplementary Table S1.

### Gene knockdown, CSR complementation, and RT-qPCR

To knock down specific genes or transcripts (such as *hs4*-eRNA) in CH12F3-2A cells, we used stealth RNAi siRNAs (Thermo Fisher) and locked nucleic acid (LNA) GapmeR antisense oligonucleotides (ASOs; QIAGEN), respectively. For each transfection, 40–80 pmol of targeting oligonucleotide was electroporated into 1 × 10^6^ cells using the Amaxa Nucleofector 96-well Shuttle System (Lonza) with the Cell Line Nucleofector Kit SF. Electroporation was performed using program #96-DI-100, optimized for CH12F3 cells. After transfection, cells were cultured for 24 h before CIT stimulation, followed by an additional 24 or 48 h of incubation. The sequences of the siRNA or ASO are listed in Supplementary Table S1.

For CSR complementation assays, co-transfection of siRNAs and plasmids encoding siRNA-resistant wild-type (WT) or mutant forms of *Hnrnpl* or *Cstf2* was performed under the same electroporation conditions. CSR efficiency was assessed by flow cytometry as described earlier. A non-targeting siRNA (siControl) was used as a negative control. Knockdown efficiency was validated by reverse transcription quantitative polymerase chain reaction (RT-qPCR) and/or immunoblotting.

For RT-qPCR analysis, total RNA was extracted using TRIzol reagent (Gibco BRL), and 1 μg of RNA was reverse transcribed using SuperScript IV Reverse Transcriptase (Thermo Fisher). qPCR was performed using PowerUp SYBR Green Master Mix and either an Applied Biosystems or Bio-Rad real-time PCR instrument. Gene expression levels were normalized to housekeeping genes, primarily *β2-microglobulin* and *Hprt*, and quantified using the 2^–ΔΔCT^ method. The primers used for qPCR are listed in Supplementary Table S1.

### Generation of *Hnrnpl* and *Cstf2* constructs

A previously described construct encoding WT hnRNPL (WT-L^R^) fused with a C-terminal 3× Flag tag [24] was used as the template for generating point and deletion mutants via site-directed mutagenesis, following the Q5 Site-Directed Mutagenesis Kit protocol (New England Biolabs, E0554). For *Cstf2*, mouse cDNA (NM_001359053.1) was amplified by RT-PCR using RNA isolated from CH12F3-2A cells. Primers containing *BamHI* and *NheI* restriction sites were used for amplification. The resulting complementary DNA (cDNA) was cloned into the pEFα-3× Flag expression vector using the In-Fusion cloning method. To render the construct resistant to siCstf2, the siRNA-targeted region was synonymously mutated without altering the encoded amino acid sequence. This siRNA-resistant WT construct (Cstf2^R^) was then used as the template to generate point and deletion mutants using the Q5 Site-Directed Mutagenesis Kit (NEB). All constructs were verified by Sanger sequencing and confirmed to express the expected proteins by immunoblotting using anti-Flag antibodies.

### Flag immunoprecipitation and proteomics sample preparation

Flag-tagged WT and mutant (F15) hnRNPL proteins were immunoprecipitated from cell lysates using anti-Flag affinity resin according to standard protocols. The immunoprecipitated complexes were resolved by sodium dodecyl sulfate–polyacrylamide gel electrophoresis (SDS–PAGE) and visualized by silver staining. The gel regions excised for mass spectrometry analysis are indicated in Fig. 3A by vertical white bars drawn over the corresponding bands for both WT and mutant samples. Excised gel slices were submitted to the Proteomics Facility at Tokushima University for mass spectrometry analysis.

### 5′ and 3′ rapid amplification of cDNA ends

Total RNA was isolated from activated CH12F3-2A cells using TRIzol reagent and assessed by spectrophotometry and gel electrophoresis. First-strand cDNA was synthesized from 1 μg of total RNA using the SMARTer RACE 5′/3′ Kit (Takara Bio) according to the manufacturer’s instructions. For 5′ rapid amplification of cDNA ends (RACE), transcript-specific primers targeting the *hs4* region were used to prime cDNA synthesis. A universal primer provided by the kit was used for PCR amplification of both 5′ and 3′ ends. Several independent clones of the RACE products were sequenced to capture the heterogeneity of the *hs4* eRNAs. Primer sequences are listed in Supplementary Table S1.

### PCR detection of LSR

LSR was assessed in CH12F3-2A cells and primary B cells following a previously established protocol with minor modifications [20]. gDNA was extracted from CH12F3-2A cells stimulated with or without CIT for 48 h and from primary splenic B cells (WT and AID-knockout) activated for 4 days with LPS and IL-4. For each condition, 100 ng of gDNA was used to set up five independent first-round PCR reactions. Touchdown PCR was performed using Herculase II Fusion DNA Polymerase (Agilent) with the following conditions: initial denaturation at 93°C for 4 min; 2 cycles at 93°C for 40 s, 64°C for 40 s, and 68°C for 4 min; 3 cycles at 93°C for 40 s, 62°C for 40 s, and 68°C for 4 min; followed by 25 cycles at 93°C for 40 s, 55°C for 40 s, and 68°C for 4 min; and a final extension at 68°C for 10 min. A nested PCR (KOD Fx Neo-Toyobo) was then performed using the first-round product as a template. Conditions were as follows: 1 cycle at 93°C for 4 min; 30 cycles at 93°C for 40 s, 64°C for 40 s, and 68°C for 4 min; followed by a final extension at 68°C for 10 min. PCR products were analyzed by 1% agarose gel electrophoresis and visualized by ethidium bromide staining. Amplification of the *Gapdh* locus served as a control for gDNA input in each sample and reaction.

### Immunoprecipitation and immunoblotting

HEK293T cells were transfected with Flag-tagged *Hnrnpl* or *Cstf2* constructs (WT or mutant) using FuGENE transfection reagent and harvested 48 h post-transfection. Transfected cells were lysed and clarified according to the instructions of the RiboCluster Profiler Kit (MBL, RN1001), in the presence of EDTA-free protease inhibitors (Roche) and RNase inhibitor (Sigma–Aldrich). For immunoprecipitation, Flag agarose beads (Sigma–Aldrich) were incubated with clarified lysates at 4°C for 4 h to overnight with rotation. Beads were then washed using the kit-recommended mild wash buffer to preserve protein–protein interactions. Bound proteins were resolved on 4%–20% SDS–PAGE gels (Bio-Rad) and analyzed by western blotting or silver staining using the SilverQuest™ Silver Staining Kit (Invitrogen). The antibodies used to detect target and co-immunoprecipitated proteins are provided in Supplementary Table S1.

### Chromatin immunoprecipitation and RNA immunoprecipitation

Chromatin immunoprecipitation (ChIP) was performed using the ChIP-IT Express Kit (Active Motif) according to the manufacturer’s protocol. Briefly, 5 × 10^6^ CH12F3-2A cells were fixed with 1% formaldehyde at room temperature, and the reaction was quenched with 0.125 M glycine. After lysis and sonication, the chromatin was sheared to an average size of 200–1000 bp. Chromatin fragments were immunoprecipitated using 3 μg of specific antibodies and eluted following the kit instructions. After reverse crosslinking, DNA was purified and analyzed by real-time PCR. Enrichment was normalized to input DNA. The antibodies and primers are listed in Supplementary Table S1.

RNA immunoprecipitation (RIP) assays were performed in CH12F3-2A cells expressing 3× Flag-tagged hnRNPL or CstF64 (WT or mutant forms). Cells (∼10^7^ per IP) were stimulated with CIT for 24 h, collected, washed, and lysed on ice in RIP lysis buffer (RiboCluster Profiler RIP Kit, MBL) supplemented with protease and RNase inhibitors. The RIP procedure was carried out using the Magna RIP Kit (Millipore) according to the manufacturer’s instructions. After the final IP wash step, TRIzol reagent was added directly to the magnetic beads to extract co-immunoprecipitated RNA. RNA was treated with DNase I and purified using the RNeasy MinElute Cleanup Kit (QIAGEN). The quality and concentration of RNA were assessed using a NanoDrop spectrophotometer or an Agilent Bioanalyzer. Reverse transcription and quantitative PCR were conducted using Power SYBR Green Master Mix (Applied Biosystems) to evaluate specific eRNA enrichment. A parallel control RIP using normal IgG was included in each experiment, following the guidelines provided by the RIP kit manufacturers (MBL and Millipore).

### CRISPR interference and pharmacological inhibition

To repress enhancer activity at the *hs4* region, we employed CRISPR interference (CRISPRi) and pharmacological inhibitors targeting distinct steps of RNA processing. For CRISPRi, small guide RNAs (sgRNAs) targeting *hs4* were designed using CRISPOR and cloned into the pSPgRNA vector (Addgene, #47108) via the *BbsI* site. The dCas9-KRAB expression plasmid (Addgene, #71236) was co-transfected with sgRNA constructs into CH12F3-2A cells using the Amaxa Nucleofector system under optimized conditions. After 24 h of incubation, cells were stimulated with CIT and cultured for an additional 24 h. Cells were then harvested for RT-qPCR or flow cytometry. A nontargeting sgRNA was used as a control.

Pharmacological perturbation of enhancer activity was performed using two small molecules with distinct mechanisms of action. Cordycepin (Sigma, C3394), a polyadenylation inhibitor, was used to assess the role of RNA 3′ end processing in *hs4*-eRNA stability. Flavopiridol (Sigma, T3652), a CDK9 inhibitor that blocks transcriptional elongation by RNAPII, was used to evaluate the effect of general transcriptional inhibition. For both compounds, multiple concentrations were initially tested to identify the optimal conditions that impaired CSR or eRNA levels while minimizing cytotoxicity. Treated cells were collected for RNA extraction and RT-qPCR as described earlier.

### Analysis of CSR junctions by nested PCR and sequencing

To analyze Sμ–Sα CSR junctions, CH12F3-2A cells transfected with siControl, si*Hnrnpl*, or si*Cstf2* (CstF64) were stimulated with CIT for 48 h. After stimulation, IgA-positive cells were isolated using anti-PE Magnetic Particles-DM (BD Biosciences), and gDNA was extracted. Switch junctions were amplified using nested PCR with primers specific to the *Sμ* and *Sα* regions, as previously described [25]. The first-round PCR was performed using PrimeSTAR HS DNA Polymerase (Takara Bio) under the following cycling conditions: 95°C for 2 min; 22 cycles of 98°C for 10 s and 68°C for 7 min. The resulting products were purified and used as templates for a second-round PCR (23 cycles) using similar conditions, with adjusted extension times as needed. PCR products between 0.3 and 1.5 kb were gel-purified, TA-tailed, and cloned into the pGEM-T vector (Promega) for Sanger sequencing. To identify the switch junctions, each sequence was aligned against the *Sμ* and *Sα* reference regions using pairwise BLAST (nBLAST). GenBank accession numbers AH005309.2 (Sμ) and D11468.1 (Sα) were used as reference sequences.

### I-SceI-based NHEJ reporter assay

The NHEJ reporter construct and the H1299dA3-1 human reporter cell line have been previously described [26, 27]. The H1299dA3-1 cells, kindly provided by Dr T. Kohno (National Cancer Center Research Institute, Tokyo), harbor an integrated NHEJ substrate that restores GFP expression upon successful repair. For siRNA-mediated gene knockdown, cells were transfected with siControl, si*HNRNPL*, or si*CSTF2*, and co-transfected with the I-*Sce*I endonuclease expression plasmid (pCBASce) using FuGENE6 transfection reagent (Roche). In some experiments, electroporation was used as an alternative method and produced comparable results. At 48 h post-transfection, cells were harvested for flow cytometry to quantify GFP-positive cells (NHEJ-repaired), and gDNA was isolated for junction analysis by PCR using primers and cycling conditions previously described [26].

As a control, the pCMV-EGFP-N1 vector (Clontech) was co-transfected with the indicated siRNAs to assess transfection efficiency. Knockdown of *HNRNPL* and *CSTF2* was validated by RT-qPCR and immunoblotting. The RT-qPCR of the integrated reporter cassette and the repair genes were conducted similarly as described earlier and normalized to *GAPDH*. The antibodies and primer sequences are available in the Supplementary Table S1.

### Cell cycle analysis by PI staining

For cell cycle analysis, H1299dA3-1 cells were transfected with si*HNRNPL*, si*CSTF2*, or siControl using the Lonza Nucleofector system (SF solution) with program #EW-127 and the Cell Line Nucleofector Kit SF. At 48–72 h post-electroporation, cells were collected, washed with PBS, and fixed in 70% ethanol at −20°C for at least 2 h. Fixed cells were washed and resuspended in PBS containing 50 μg/mL PI and 100 μg/mL RNase A. After incubation at 37°C for 30 min in the dark, samples were analyzed by flow cytometry. Cell cycle profiles were determined using FlowJo software (BD Biosciences), with gating strategies applied to exclude debris and doublets based on forward/side scatter and pulse-width discrimination.

### CH12F3-2A cell proliferation assay

To assess the effect of *Hnrnpl* or *Cstf2* depletion on CH12F3-2A cell proliferation, cells were labeled using the Cell Trace Violet CFSE Cell Proliferation Kit (Thermo Fisher) according to the manufacturer’s instructions. Briefly, cells were stained with 5 μM CFSE for 15 min at 37°C, washed, and cultured for 24 h. Experimental timelines and other details, including CIT stimulation, are shown in the corresponding figure panels. CFSE dilution was monitored by flow cytometry in *hnRNPL*-depleted cells (siRNA) compared with siControl and in *Cstf2* KO cells compared with WT CH12F3-2A cells. As a positive control for proliferation inhibition, WT cells were treated with 2 μg/mL aphidicolin.

## Result

### The vRRM2 and vRRM3 domains of hnRNPL play a critical role in CSR

**Figure 1.**
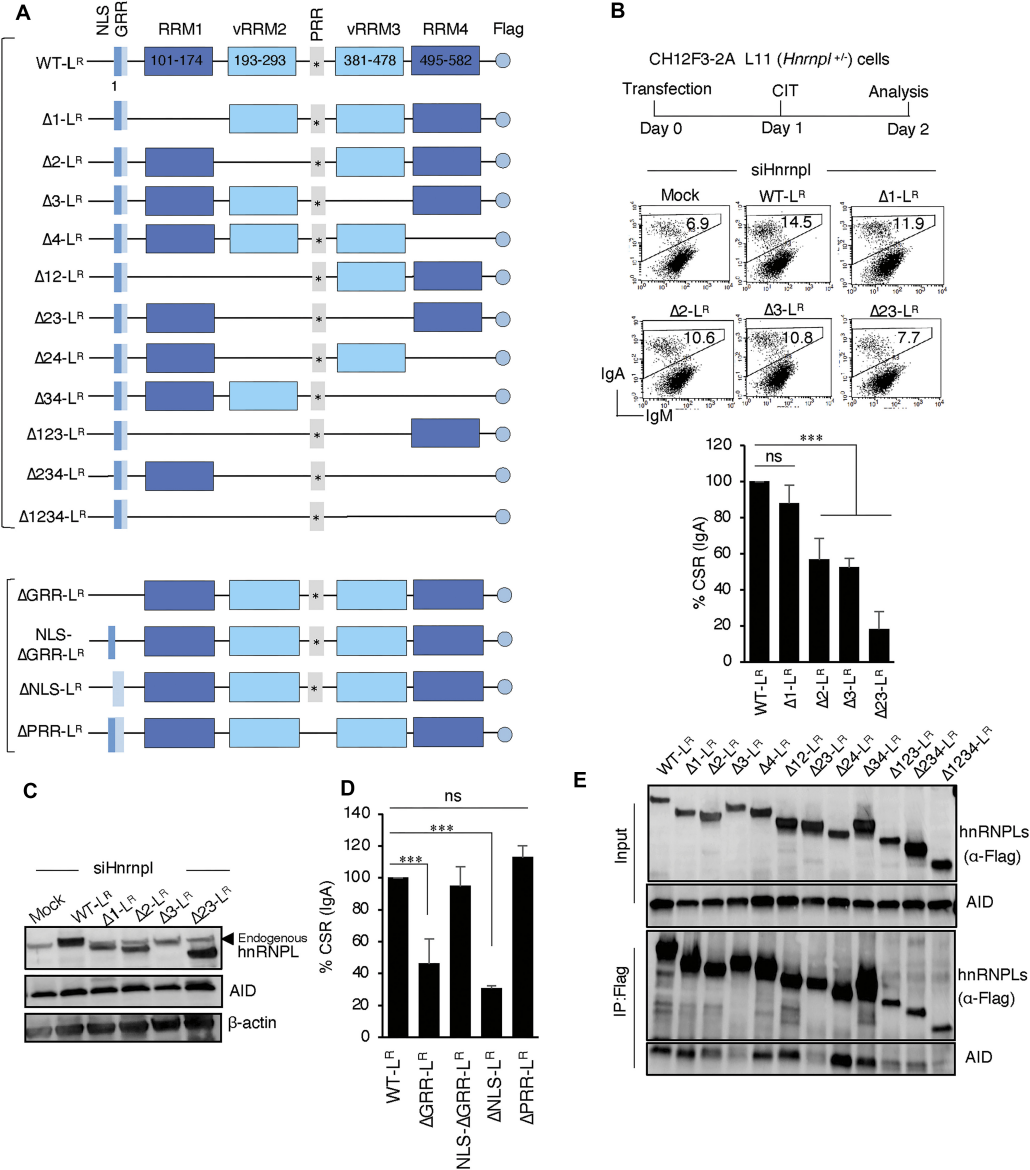
The specific RRM domains of hnRNPL are essential for CSR. (**A**) Schematic representation of the hnRNPL protein and its domain deletion mutants. The various domains and RRMs (RNA recognition motifs) are indicated. The FLAG epitope is located at the C-terminus. The star (*) indicates the position of siRNA resistance mutagenesis. (**B**) The schematic of the experimental timeline in CH12F3-2A L11 cells (top). Representative FACS profiles showing CSR complementation efficiency by WT-hnRNPL (WT-L^R^) and its domain deletion mutants are indicated (middle). The number in each FACS plot represents the percentage of IgA. The data are presented as mean ± SEM from three independent experiments in the bar blot (bottom). (**C**) Western blots depicting the expression of various hnRNPL proteins and AID in cells transfected with sihnRNPL or mock control. The antibodies used are indicated next to the respective panel. (**D**) Quantification of CSR efficiency in cells expressing WT hnRNPL or its N-terminal-specific mutants. Data are presented as mean ± SEM from three independent experiments. (**E**) Co-IP experiment to assess the interaction between vRRM domains of hnRNPL and AID. Cell lysates were immunoprecipitated with anti-Flag antibody, and the immunoprecipitates were analyzed with the indicated antibodies. Data are presented as mean ± SD from two or three independent experiments (*n* = 2–3). Statistical analysis was performed using Student’s *t*-test (***P ≤ .001; ns, non-significant).

In our previous study, we broadly examined the significance of the RNA-binding domains (RRMs) of hnRNPL in CSR [18]. To develop a deeper understanding, we employed a more detailed mapping approach to pinpoint the crucial functional region in the hnRNPL–RRMs. While the RRM domain follows a typical β1α1β2β3α2β4 topology, a variant RRM (vRRM) domain contains an additional β5 strand, resulting in a distinct β1α1β2β3α2β4β5 topology [28, 29]. Among the four RRMs, RRM2 and RRM3 are of the vRRM type (Fig. 1A), featuring the tripartite VRC motif that facilitates interaction with CA-rich repeat RNAs [24].u To investigate the vRRM’s function, we analyzed the CSR complementation efficiency of RRM deletion mutants compared to the WT in the hnRNPL-depleted mouse B cell line (CH12F3; Clone 2A). The constructs were created to express transcripts that are resistant to siRNA (siHnrnpl)-mediated degradation and thus were designated as “R” (Fig. 1A–C). The CH12F3-2A line was also engineered to overexpress *Bcl2*, providing robust viability when subjected to gene knockdown. Following transfection, the CH12F3-2A cells efficiently switch from IgM to IgA within 24 h of CIT (CD40L, IL-4, and TGFβ) stimulation. The expression of WT Hnrnpl (L-WT^R^) fully restored CSR, but the deletion of vRRM2 (Δ2-L^R^) or vRRM3 (Δ3-L^R^) only partially rescued CSR (Fig. 1B). As expected, a combined deletion of the two vRRMs did not complement CSR, indicating that these two domains are particularly crucial for CSR. We confirmed the knockdown of endogenous hnRNPL and expression of the ectopically expressed hnRNPL^R^ constructs (Fig. 1C). Notably, the deletion of the N-terminal short GRR domain affected CSR, which is attributable to the loss of the nuclear localization signal (NLS) embedded within. We validated that NLS deletion impairs CSR and that supplementing NLS in ΔGRR can fully restore CSR (Fig. 1A and D). In contrast, deleting the proline-rich PRR domain in between the two vRRMs had no discernible impact on CSR, further emphasizing the significance of a localized region within the vRRMs. Co-immunoprecipitation (Co-IP) analysis in HEK293T cells revealed that the two vRRM domains, particularly vRRM3, primarily interact with AID (Fig. 1E).

### Identification of the loss-of-CSR function region within the vRRMs

**Figure 2.**
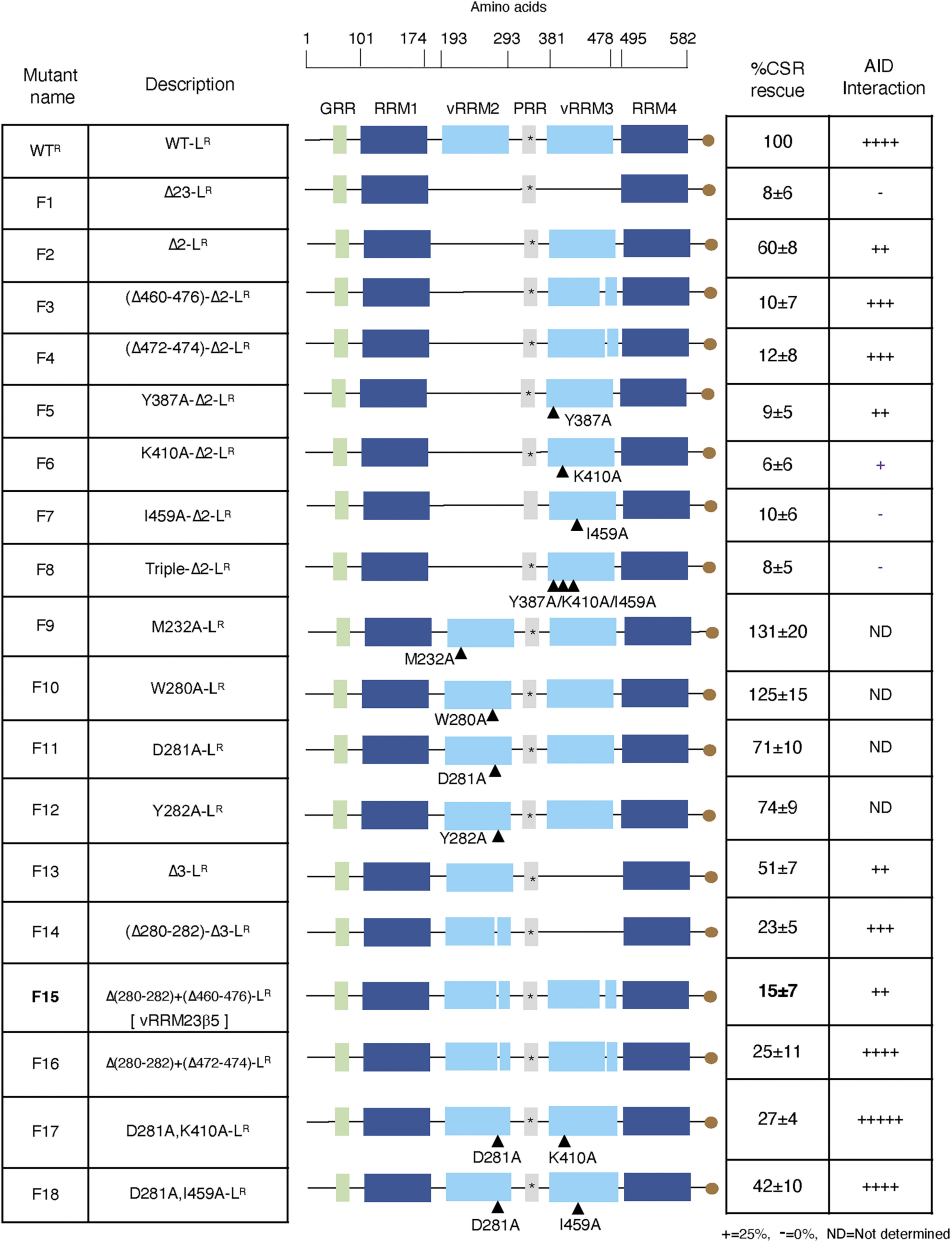
Fine mapping of hnRNPL vRRMS and its relation to CSR and AID interaction. The hnRNPL vRRM2 and vRRM3 are essential for optimal CSR; deletion of either vRRM2 or vRRM3 significantly reduces CSR efficiency. The specific residues within vRRM2 and vRRM3 are important: mutations in key amino acid residues within vRRM2 and vRRM3 (e.g. F5, F6, F7, F11, F12, F15, F17, and F18) affect CSR efficiency. The F15 is a critical CSR loss-of-function mutant, harboring patches of deletion in vRRM2 and vRRM3, which disrupts the β5-strand, a feature unique to the vRRMs. The combined defect significantly impaired CSR efficiency.

To assess the importance of each vRRM, we generated a series of mutants (F3–F12 and F14–F18) that involved point mutations and/or deletions, aimed at disrupting the unique β5 strand and the VRC motif (Fig. 2). For example, to eliminate a distinctive feature in vRRM3, we deleted 17 amino acids (Δ460–476) that encompass the VRC (F3) on Δ2-L^R^ backbone (F2). Removing these residues in vRRM3 significantly reduced the CSR rescue efficiency compared to Δ2-L^R^ (F2 versus F3), without compromising their interaction with AID. Additionally, mutating three residues (Y387A, K413A, and I459A) involved in stabilizing vRRM3 hampered CSR rescue efficiency (mutants F5–F8).

Mutating several conserved residues on vRRM2 (M232A, W280, D281, and Y282) in a WT (WT-L^R^) backbone did not significantly affect CSR (F9–F12). However, disrupting the β5 strand in vRRM2 by deleting residues W280–Y282 on the Δ3-L^R^ backbone decreased CSR rescue efficiency (F14). Subsequently, we generated the F15 mutant (vRRM23β5) by combining β5 strand-specific deletions in vRRM2 (Δ280–Y282) and vRRM3 (Δ460–476), resulting in a significantly defective CSR. A closely related F16 mutant also exhibited reduced CSR, highlighting the crucial role of the β5 strand unique to vRRM2 and vRRM3 in CSR. Furthermore, combinatorial mutations involved in stabilizing vRRM23 showed similar reductions in CSR (F17–F18). Point mutations of D281A and Y282A, but not D281A alone, moderately affected CSR, again suggesting the requirement of both vRRM2 and vRRM3. Despite varying degrees of CSR defects, AID was not entirely dissociated from any of the mutants, suggesting an AID-interaction-independent role of hnRNPL–vRRM in CSR.

### hnRNPL–CstF64 interaction requires the structural integrity of the vRRMs

**Figure 3.**
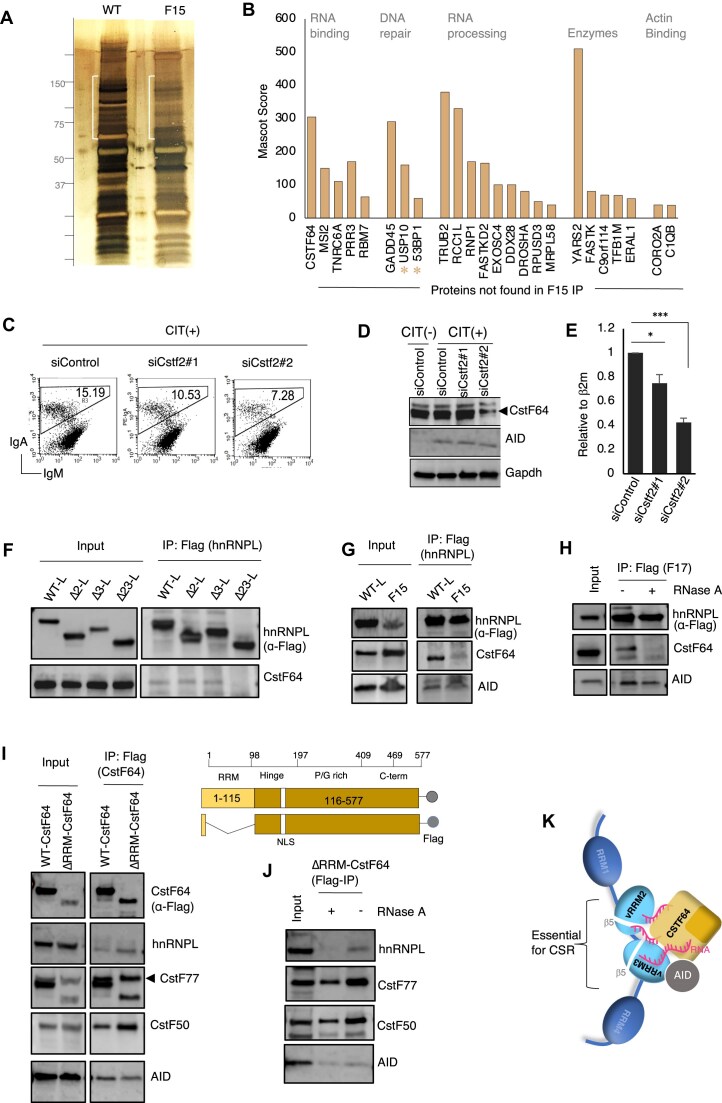
CstF64 interacts with hnRNPL vRRMs and is required for CSR. (**A**) Comparison of co-IP analysis between WT and a CSR loss-of-function mutant, F15. Silver-stained gel showing that several proteins fail to bind to the vRRM23β5 domain defective F15 mutant. The white bars indicated the region sliced for MS analysis. (**B**) Proteomic analysis reveals proteins missing in the F15 mutant compared to the WT. (**C**–**E**) Flow cytometry analysis of CSR efficiency in CH12F3-2A cells following *Cstf2* (CstF64) knockdown (C). The siCstf2#2 oligo demonstrated the highest knockdown efficiency in the western blot (D), with the arrowhead marking the correct size of the CstF64 protein. Knockdown efficiency verification by RT-qPCR (E); data are presented as mean ± SD from independent experiments (*n* = 2–3). Statistical analysis was performed using Student’s *t*-test (*P ≤ .05; ***P ≤ .001; ns, non-significant). (**F**–**H**) Immunoprecipitation analysis of WT and hnRNPL vRRM domain mutants in 293T cells. Loss of vRRM2 and vRRM3 in hnRNPL fails to pull down HEK293T CstF64 (F). IP analysis of WT-hnRNPL F15 mutant with defective vRRM2 and vRRM3, which does not interact with CstF64 (G). IP analysis of WT-hnRNPL and F17 mutant, in the presence and absence of RNase, shows that the interaction between CstF64 and hnRNPL is RNA-dependent (H). (**I**, **J**) The schematic represents C-terminally Flag-tagged CstF64 and functional elements in it. Immunoprecipitation analysis of WT and a CstF64 mutant, lacking the single RRM domain at the N-terminus, in HEK293T cells (I). IP analysis of the CstF64 mutant in the presence and absence of RNase A (J). (**K**) An illustration of hnRNPL and CstF64 complex, highlighting the importance of vRRM2 and vRRM3 of hnRNPL for CSR, which provides potential interaction surface for interacting proteins and RNA.

To identify critical proteins dissociating from the hnRNPL–vRRM mutant, we compared the protein–protein interaction profile between WT and F15 mutant (vRRM23β5). For this purpose, we expressed FLAG epitope-tagged WT and F15 hnRNPL in HEK293T cells, and the IPed proteins were analyzed by gel electrophoresis and silver staining. MS analysis was performed on the specified regions of the gel, which exhibited a significant difference between the WT and the mutant, as shown in Fig. 3A. We identified 24 proteins, including 53BP1, that were specifically missing from the F15 mutant IP (Fig. 3B and Supplementary Fig. S1C). Identifying 53BP1 was relevant given hnRNPL’s predicted involvement in CSR through DNA repair [18, 24]. We also identified the deubiquitinase protein Usp10, which has recently been shown to be involved in CSR by directly interacting with AID [30].

To identify relevant novel interacting partners of hnRNPL vRRM, we knocked down putative candidates that did not interact with the F15 mutant (Fig. 3B and Supplementary Fig. S1A). The polyadenylation/cleavage stimulating factor CstF64 was a promising candidate whose depletion affected CSR. Knockdown of the *Cstf2* gene (siRNA#2) showed 50% knockdown efficiency with comparable CSR impairment (Fig. 3C–E). We confirmed consistent and reproducible interaction between CstF64 and hnRNPL (Fig. 3F and Supplementary Fig. S1B). The hnRNPL interacted with CstF64 through both vRRMs, and thus, deleting one vRRM could not completely disrupt their interaction. The failure of CstF64 pull-down by F15(vRRM23β5), where β5 strand in vRRM2 and vRRM3 was disrupted (Fig. 3G), further confirms the result and is in line with the absence of CstF64 in the F15 mutant MS analysis (Fig. 3B). Interestingly, the F17 mutant, which carries point mutations D281A and K410A in vRRM2 and vRRM3, respectively, was able to retain some interaction with CstF64 but dissociated upon RNase A treatment (Fig. 3H). This implies that unidentified RNA species can potentiate the interaction between hnRNPL and CstF64.

Reciprocal IP analysis revealed that both wild-type (WT-CstF64) and RRM-deleted mutant (ΔRRM-CstF64) could pull down hnRNPL, suggesting the RRM domain of CSTF64 does not contribute to the interaction with hnRNPL (Fig. 3I). However, ΔRRM-CstF64 showed an RNA-dependent interaction with hnRNPL (Fig. 3J), suggesting the hnRNPL–vRRMs and the associated RNA contributed to the interaction, as observed in Fig. 3H and G. The presence of other CstF subunits like CstF77 and CstF50 confirms their direct interaction with CstF64 and the specificity of the IP. Altogether, we validated hnRNPL’s interaction with CstF64 through the vRRMs with unique β5 structures (Fig. 3K).

### CstF64 promotes CSR and 3′RR eRNA transcription similar to hnRNPL

**Figure 4.**
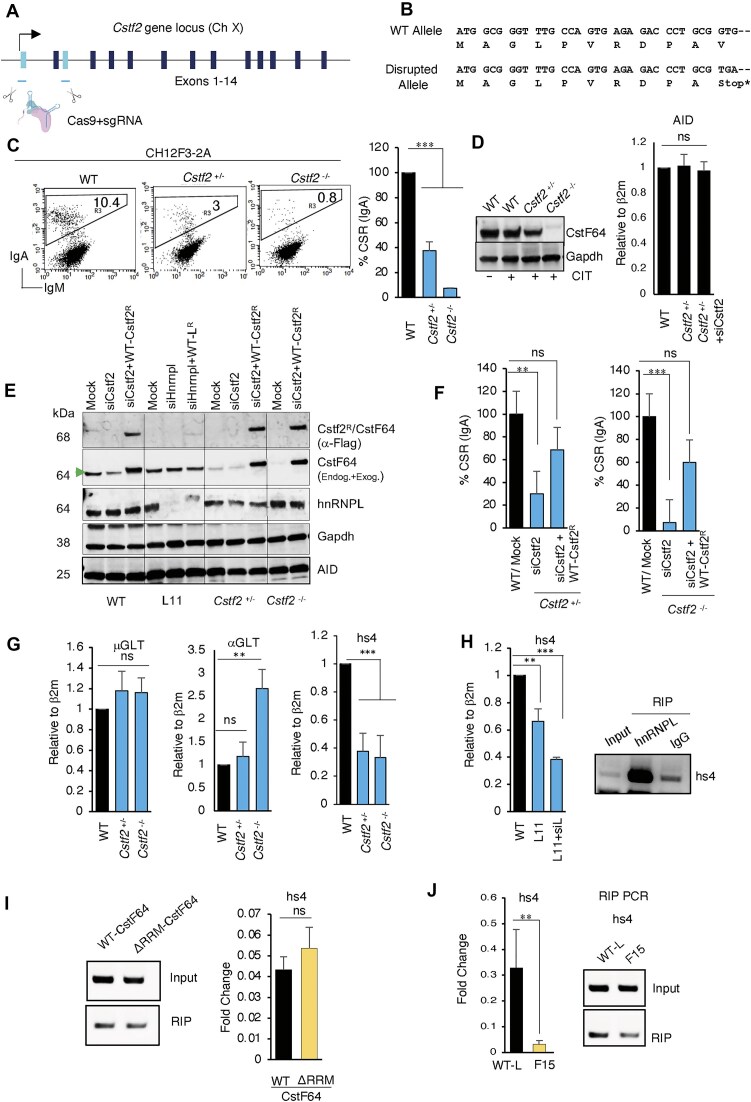
hnRNPL regulates CSR via CstF64 and eRNA. (**A**, **B**) Generation of *Cstf2* KO CH12F3-2A cells. Schematic of the *Cstf2* gene locus and the CRISPR–Cas9 strategy used to disrupt *Cstf2* (A). Two gRNAs, designed to target exon-1 and exon-3, are indicated by cyan bars below the exons. The position of a premature termination codon, confirmed by sequencing, on the disrupted allele is shown (B). (**C**, **D**) Analysis of heterozygous and homozygous *Cstf2* KO CH12F3-2A clones. Representative FACS profiles and compilation of independent experiments as a bar plot (C). Western blots showing expression of CstF64 protein in heterozygous and homozygous KO CH12F3-2A cells, followed by RT-qPCR of AID mRNA expression (D). (**E**) Western blot analysis showing the knockdown of endogenous CstF64 and hnRNPL and overexpression of siRNA-resistant WT Cstf2 (Cstf2^R^) and WT hnRNPL (WT-L^R^). Exogenous proteins were detected by anti-Flag antibody. The green arrow indicates the endogenous CstF64. (**F**) Bart plots summarize independent experiments of CSR rescue efficiency by overexpressing WT CstF64 from the Cstf2^R^ construct in the presence and absence of siCstf2 in *Cstf2*-deficient CH12F3-2A cells. (**G**) CstF64-deficient cells exhibit unperturbed switch germline transcription but show impairment in hs4 enhancer transcription. Bar plots present the RT-qPCR analysis from independent experiments. (**H**) RT-qPCR data showing hnRNPL KD reduces the hs4-eRNA transcription, followed by specific enrichment of hs4-eRNA in hnRNPL-RIP but not in IgG-RIP, a negative control. (**I**) RT-qPCR data of RIP demonstrate no specific enrichment of hs4-eRNA between WT and RRM-deleted CstF64. (**J**) RIP RT-qPCR analysis of hnRNPL WT and F15 mutant showing that vRRMs are required for hs4-eRNA-mediated interaction of hnRNPL with CstF64. The data presented as bar plots are mean ± SD from two or three independent experiments (*n* = 2–3). Statistical analysis was performed using Student’s *t*-test (*P ≤ .05; **P ≤ .01; ***P ≤ .001; ns, non-significant).

To demonstrate unequivocally that CstF64 is required for CSR, we disrupted *the Cstf2* gene locus by CRISPR/Cas9 targeting exon 1 and exon 3 in CH12F3-2A cells, which resulted in an early stop codon (Fig. 4A and B). We successfully obtained heterozygous (*Cstf2*^+/−^) as well as homozygous (*Cstf2*^−/−^) KO clones (Supplementary Fig. S2A–C). We observed more than a 50% reduction in CSR for the *Cstf2*^+/−^ clone, while a more significant impairment of CSR was noted in the case of *Cstf2*^−/−^ clone, confirming the specific requirement of CstF64 (Fig. 4C and D). We verified the restoration of CSR in *Cstf2*^+/−^ and *Cstf2*^−/−^ clones by stably expressing WT-*Cstf2*^R^ (Fig. 4E), which ensured the gene targeting and CSR restoration specificity for CstF64 (Fig. 4F).

To understand the mechanism, we first examined the effect of CstF64 disruption on switch germline transcription by RT-qPCR. We found that μGLT and αGLT from donor and acceptor S regions were unperturbed in *Cstf2*^+/−^ and *Cstf2*^−/−^ cells, although αGLT was modestly elevated in *Cstf2*^−/−^ cells (Fig. 4G). Surprisingly, however, transcription of hs4 eRNAs from the IgH 3′RR, which plays a pivotal role in CSR [10], was markedly reduced in both *Cstf2*^+/−^ and *Cstf2*^−/−^ cells. Similarly, we also observed a comparable level of eRNA downregulation in L11 cells (hnRNPL^+/−^) transfected with siHnrnpl (Fig. 4H).

Next, we performed RIP analysis to examine whether the 3′RR eRNA binds to or forms a complex with hnRNPL and CstF64 in B cells undergoing CSR. Indeed, we could detect the 3′RR-specific eRNA by PCR in hnRNPL-RIP, which was significantly enriched in hnRNPL-RIP compared to control IgG-RIP (Fig. 4H). As predicted, we recovered 3′RR-eRNA from both WT-CstF64 and ΔRRM-CstF64 RIP, which was further confirmed by RT-qPCR (Fig. 4I). The finding was in line with the observation that the RNA-dependent interaction between CstF64 and hnRNPL is mainly through vRRMs of the hnRNPL (Fig. 3K). Accordingly, no eRNA was detected in the RIP analysis of mutant F15 compared to WT hnRNPL (Fig. 4J). We did not analyze transcripts from hs1.2 and other hs regions in the RIP samples; such possibilities cannot be ruled out. However, based on the results, we conclude that hnRNPL forms a complex with CstF64, along with the hs4 eRNA, and also enhances the transcription of these eRNAs from the hs4-IgH 3′RR.

### The canonical function of CstF64 is dispensable for 3′RR eRNA regulation

**Figure 5.**
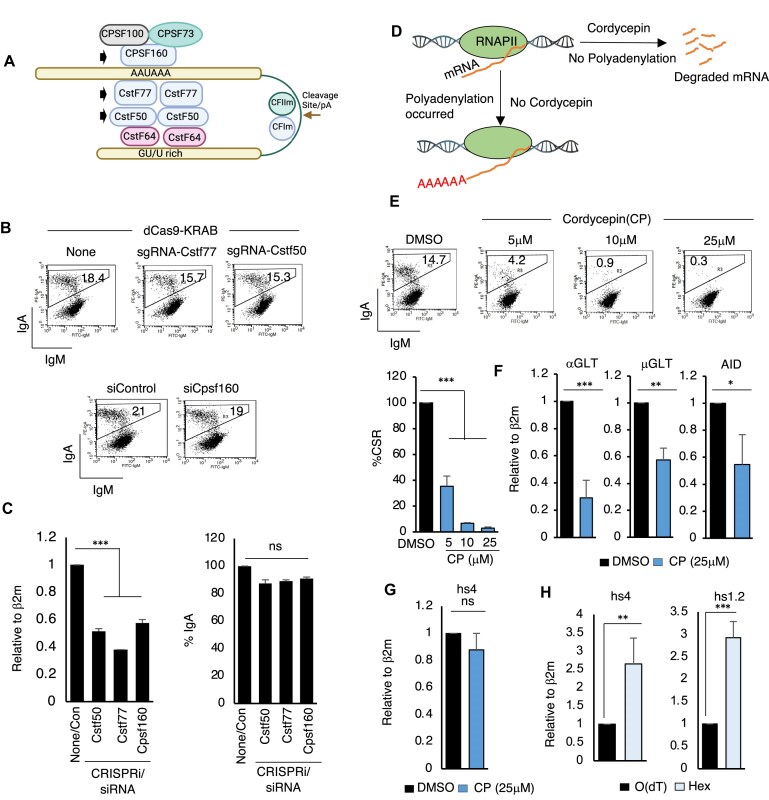
CstF64 regulates CSR independently of eRNA processing. (**A**) Schematic representation of the canonical role of the CstF complex in RNA maturation. (**B**) FACS analysis showing the CSR efficiency in siRNA or CRISPRi(dCas9-KRAB)-mediated downregulation of CstF64-associated protein components. (**C**) RT-qPCR analysis confirms the effectiveness of siRNA or CRISPRi-mediated downregulation of CstF subunits, followed by a CSR analysis profile of the respective treated cells as indicated. (**D**) Schematic representation of the mechanism of action of cordycepin (CP), a potent pharmacological inhibitor of polyadenylation. Since CP was dissolved in DMSO, an equivalent amount of DMSO, present in the highest CP treatment, served as a control. (**E**) FACS analysis demonstrating the effect of CP on CSR at varying concentrations in CH12F3-2A cells. (**F**) RT-qPCR analysis of CP (25 μM)-treated CH12F3-2A cells on GLTs and AID expression. (**G**) RT-qPCR analysis showing the ineffectiveness of CP on hs4-eRNA processing. (**H**) RT-qPCR analysis showing the enrichment of non-polyadenylated over polyadenylated eRNA from 3′RR. The DNase-treated RNA from activated CH12F3-2A cells was reverse transcribed with either oligo (dT) or random hexamer (Hex). All data are presented as mean ± SD from two or three independent experiments (*n* = 2–3). Statistical analysis was performed using Student’s *t*-test (***P ≤ .001; ns, non-significant).

Since CstF64, together with CstF50 and CstF77, is known to assist the CPSF complex in cleavage/polyadenylation of mRNA in mammalian cells [31] (Fig. 5A), we wondered whether the disruption of this function is the underlying cause of the CSR defect in CstF64-deficient CH12F3-2A cells.

To investigate such a possibility, we first planned to deplete the two direct interacting partners of CstF64, CstF50, and CstF77. We used CRISPRi, where the transcriptional repressor KRAB fused to catalytically inactive Cas9 (dCas9) was delivered to the target gene promoter through an sgRNA to suppress CstF50 and CstF77 expression. However, we did not observe any change in the CSR efficiency after transfection of sgRNA targeted to CstF50 or CstF77 promoter in KRAB-dCas9-expressing CH12F3-2A cells (Fig. 5B, top). We also knocked down *Cpsf1*(CPSF160), the largest subunit of the CPSF complex, which recognizes the poly(A) signal sequence in 3′UTR and helps in cleavage, polyadenylation, and stabilizing the complex [31]. Again, we did not observe any significant inhibition of CSR in siCpsf1-transfected CH12F3-2A cells (Fig. 5B, bottom). Although we confirmed the effectiveness of CRISPRi and siCpsf1 by estimating the transcript level of CstF50, CstF77, and CPSF160 by RT-qPCR (Fig. 5C) and protein level by WB (Supplementary Fig. S2D), the suppression level did not reflect on the CSR efficiency.

We reasoned that the reduced or lack of expression of a single subunit of a multi-subunit complex, such as CstF/CPSF, may not drastically affect global RNA processing or the stability of the cleavage/polyadenylation complex, which may lead to impaired CSR. Therefore, we embarked on using a pan-polyadenylation inhibitor, cordycepin (CP) [32], an analog of adenosine, which has a proven record of exerting a global 3′ pre-mRNA processing defect (Fig. 5D). We treated CH12F3-2A cells with different concentrations of CP and monitored CSR and expression of crucial transcripts associated with CSR, which include GLTs, AID, and the eRNAs from hs4-IgH 3′RR. As expected, CP treatment significantly decreased CSR at all tested concentrations compared to the control DMSO (Dimethyl Sulfoxide), with notable inhibition of GLT and AID expression (Fig. 5E and F).

Surprisingly, we did not observe any change in the hs4-eRNA transcription (Fig. 5G), even though enhancer-transcribed eRNAs have been reported to be comprised of both poly(A) and non-poly(A) transcripts [33]. To confirm this is the case for IgH 3′RR-derived hs4 eRNAs, we conducted 3′ and 5′ RACE using gDNA-free total RNA from CIT-activated CH12F3-2A cells. Numerous transcripts, with or without poly(A) and ranging from 200 to 500 bp, were identified. Admittedly, the RACE analysis has limitations, and we cannot fully grasp the complete nature of the transcripts due to their heterogeneous characteristics and bi-directional transcription. Furthermore, little is known about the structure-function relationship of 3′RR eRNAs in IgH super-enhancer activation involving RNAPII elongation. Considering the complexity and the limited information, the RACE-derived products we analyzed here are designated as “hs4-crossing transcripts” in Supplementary Fig. S3.

Nevertheless, the results prompted us to examine the relative abundance of poly(A) versus non-poly(A) eRNA produced from the IgH 3′RR. We, therefore, isolated total RNA from CSR-activated CH12F3-2A cells, treated them with RNase-free DNase, and cleaned them up by column purification. The RNA with a high RIN value was reverse transcribed by Oligo(dT) or random hexamer and subjected to qPCR. We detected higher eRNA amplification in random hexamer-primed cDNA compared to Oligo(dT)-primed cDNA from independent RNA sources, suggesting a significant population of 3′RR hs4-eRNAs from the IgH locus lack poly(A) (Fig. 5H), which is consistent with the published report [34]. No eRNA was detected in the negative control sample, and the depletion of hnRNPL/CstF64 did not affect the relative ratio (not shown).

### hnRNPL/CstF64 complex promotes hs4 transcription through RNAPII-S2P

**Figure 6.**
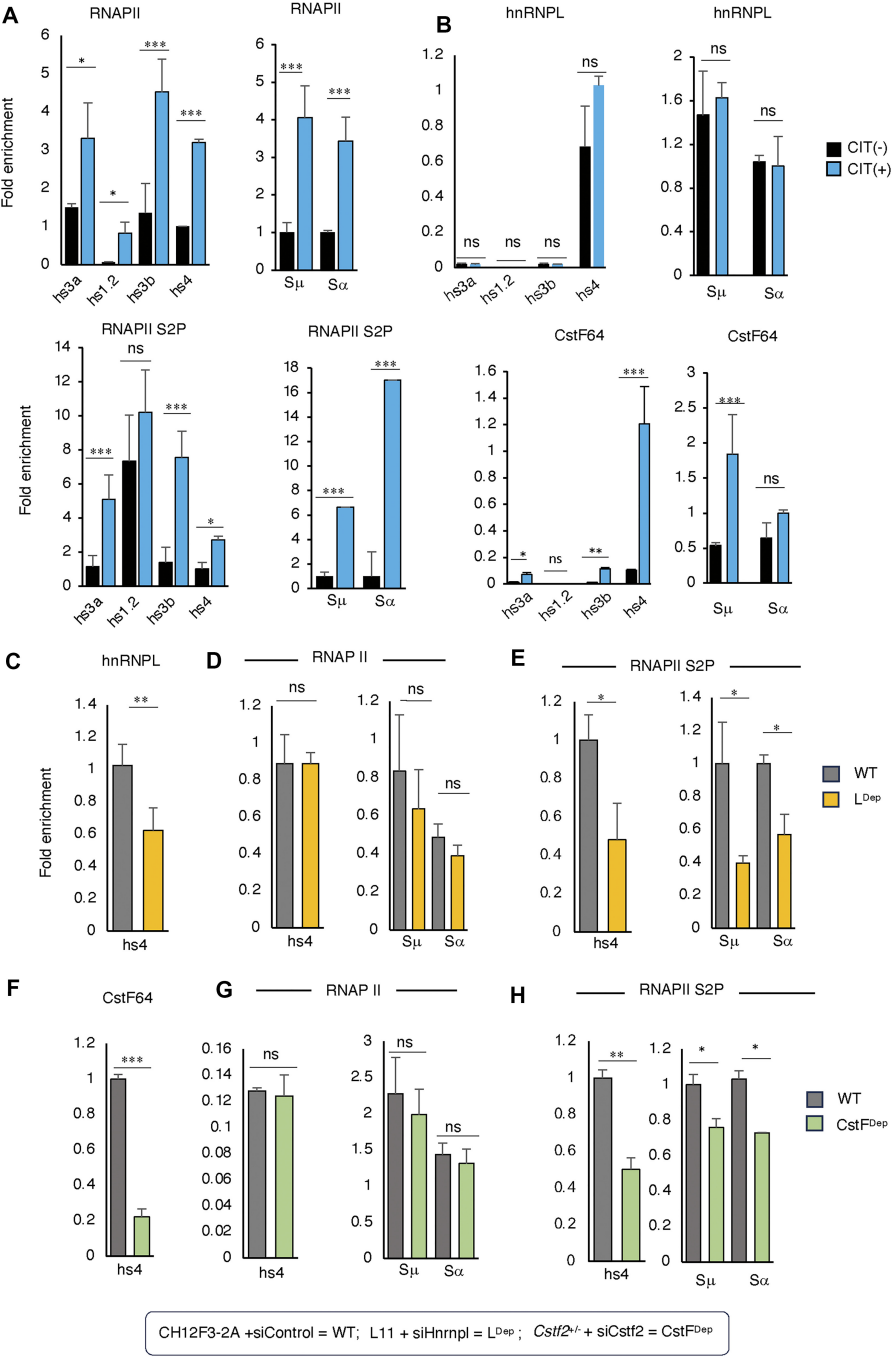
CstF64 and hnRNPL facilitate 3′RR eRNA elongation. (**A**, **B**) ChIP-qPCR analysis demonstrates the enrichment of RNAPII, its elongating form (S2P), hnRNPL, and CstF64 at the 3′RR enhancer and S regions in CH12F3-2A cells. The experiment was performed both with [CIT (+)] and without [CIT (−)] stimulation. (**C**–**E**) ChIP-qPCR analysis shows reduced occupancy of hnRNPL (C) and RNAPII S2P (E) at the 3′RR enhancer and S region following hnRNPL depletion (L^Dep^). The total RNAPII occupancy remains unaffected (D). (**F**–**H**) ChIP-qPCR analysis shows reduced occupancy of CstF64 (F) and RNAPII S2P (H) at the 3′RR enhancer and S region following CstF64 depletion (Cst^Dep^). The total RNAPII occupancy remains unaffected (G). All ChIP data were normalized to the DNA input signals, followed by the maximum value in each dataset. The data are presented as mean ± SD from three or four independent experiments (*n* = 3–4). Statistical analysis was performed using Student’s *t*-test (*P ≤ .05; **P ≤ .01; ***P ≤ .001; ns, non-significant).

The transcriptional down-modulation of 3′RR eRNA in hnRNPL or CstF64 deficiency suggests a role beyond the canonical function of CstF64 in the cleavage polyadenylation complex. We hypothesized that the hRNPL–CstF64 complex might associate with the transcription machinery at 3′RR, influencing eRNA transcription. In CIT-stimulated CH12F3-2A cells, we observed high RNAPII occupancy, including the elongating form (RNAPII-S2P), at the 3′RR and S regions (Fig. 6A). As predicted, we also detected the enrichment of CstF64 in the S regions and 3′RR, especially at the hs4 locus (Fig. 6B), but not hnRNPL. Overall, CstF64 showed a similar enrichment profile as observed for RNAPII-S2P (Fig. 6A and B). This observation is consistent with the existing reports indicating that CstF interacts with pre-mRNA and RNAPII-CTD [35, 36].

Strikingly, depletion of hnRNPL decreased RNAPII-S2P occupancy at 3′RR and S regions (Fig. 6C–E). We did not observe any change in the enrichment of total RNAPII, suggesting that hnRNPL is involved in transcription elongation but not required to recruit RNAPII at the 3′RR and S regions. Similarly, CstF64 depletion also led to decreased occupancy of RNAPII-S2P at 3′RR and S regions (Fig. 6F and H). We did not observe any change in the enrichment of hnRNPL or total RNAPII, suggesting that CstF64 is involved in transcription elongation but not required for the recruitment of RNAPII or hnRNPL at the 3′RR and S regions. Based on these findings, the hnRNPL–CstF64 complex is essential for optimal RNAPII-S2P formation at 3′RR and S regions. The presence of the hnRNPL–CstF64 complex at the 3′RR may explain its requirement for full-length eRNA synthesis.

**Figure 7.**
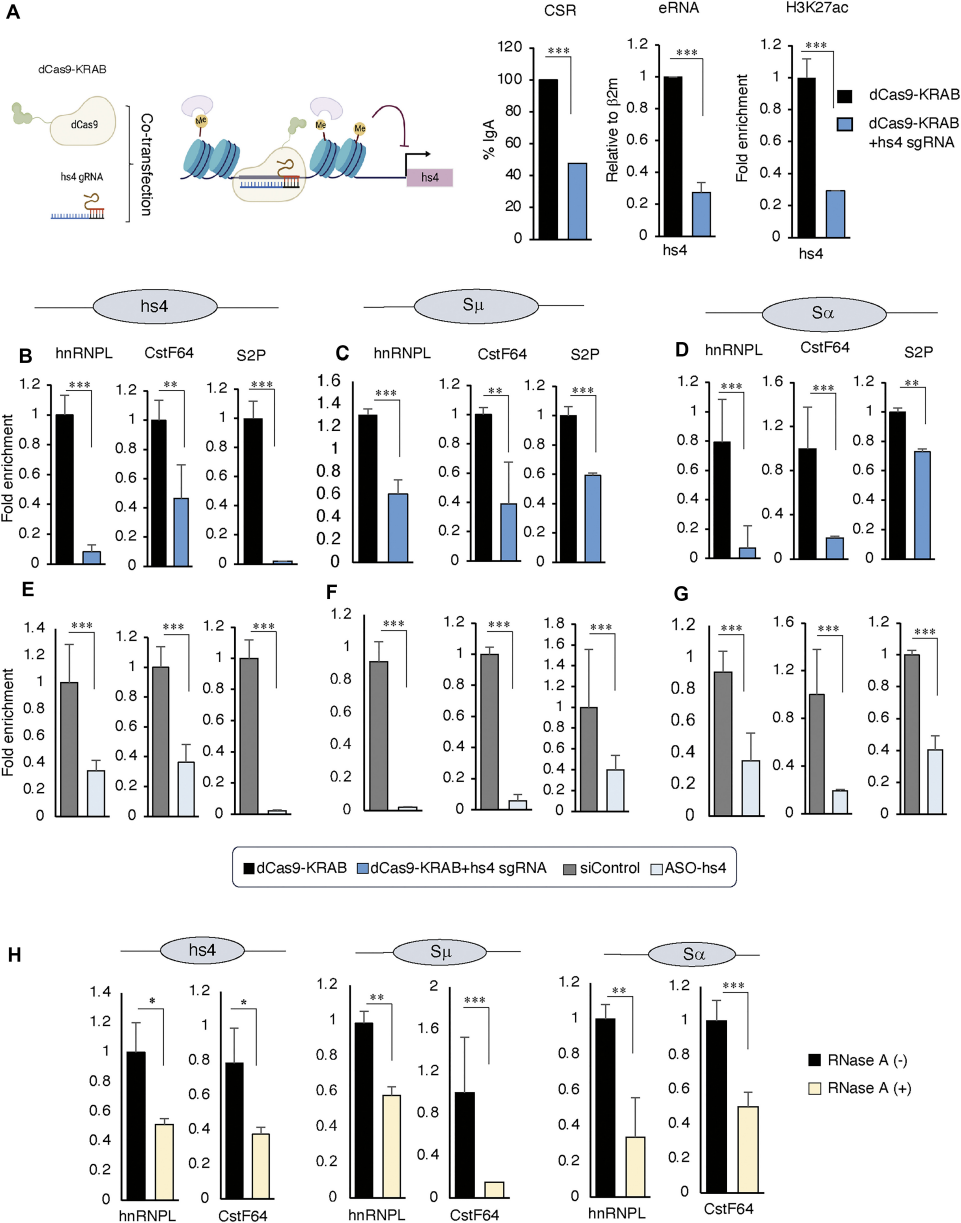
Suppression of hs4-eRNA decreases elongating RNAPII, hnRNPL, and CstF64 at the IgH locus. (**A**) Schematic representation of dCas9-KRAB (CRISPRi) targeting of the hs4 enhancer for transcriptional repression. gRNA and dCas9-KRAB were co-transfected into CH12-F3-2A cells. The KRAB domain of CRISPRi induces repressive histone methylation marks around the target site. Targeting hs4 with dCas9-KRAB leads to downregulation of hs4 transcription, marked by reduced H3K27ac levels (an enhancer activation marker), along with decreased hs4-eRNA transcription and reduced CSR. Cells transfected with dCas9-KRAB alone served as a negative control. (**B**–**D**) ChIP-qPCR analysis showing reduced recruitment of hnRNPL, CstF64, and RNAPII S2P to the hs4 enhancer, Sμ, and Sα upon hs4-eRNA inactivation by CRISPRi. (**E**–**G**) ChIP-qPCR analysis showing reduced recruitment of hnRNPL, CstF64, and RNAPII S2P to the hs4 enhancer, Sμ, and Sα upon hs4-eRNA inactivation by ASO. (**H**) ChIP-qPCR shows the enrichment of hnRNPL and CstF64 in the presence and absence of RNase A at the hs4 enhancer, Sμ, and Sα. The reduced occupancy of hnRNPL and CstF64 due to RNase may indicate an RNA-mediated (presumably eRNA and/or unknown noncoding RNAs) interaction of the complex with the IgH locus chromatin. Data are presented as mean ± SD from two or three independent experiments (*n* = 2–3). Statistical analysis was performed using Student’s *t*-test (*P ≤ .05; **P ≤ .01; ***P ≤ .001; ns, non-significant).

To test the involvement of eRNA or enhancer activation on the axis of hnRNPL–CstF64 and RNApoll-S2P, we inactivated the 3′RR enhancer by CRISPRi technology. The transfection of hs4-specific sgRNA in dCas9-KRAB expressing CH12F3-2A cells reduces the hs4 eRNA transcription and concomitantly H3K27ac and CSR (Fig. 7A) as previously observed [10]. Strikingly, a marked decrease of hnRNPL and CstF64 occupancy was observed in the inactivated enhancer, along with reduced occupancy of RNAPII-S2P (Fig. 7B). As we previously noticed that eRNA suppression at 3′RR also affects the S region histone epigenetics and protein recruitment, we also observed diminished occupancy of hnRNPL, CstF64, and RNAPII-S2P in the S regions (Fig. 7C and D). Direct depletion of hs4 eRNA transcripts by locked nucleic acid-modified antisense oligo (LNA-ASO) also confirmed the decreased occupancy of hnRNPL, CstF64, and RNAPII-S2P at 3′RR and S regions (Fig. 7E–G). Consistently, the occupancy of hnRNPL and CstF64 at the IgH locus is also sensitive to RNase A treatment (Fig. 7H). Taken together, we conclude that transcriptional suppression or degradation of hs4-eRNA by CRISPRi or ASO diminishes the occupancy of the hnRNPL–CstF64 complex involved in RNAPII-S2P formation required for eRNA transcription.

### Involvement of hnRNPL–CstF64 in LSR

**Figure 8.**
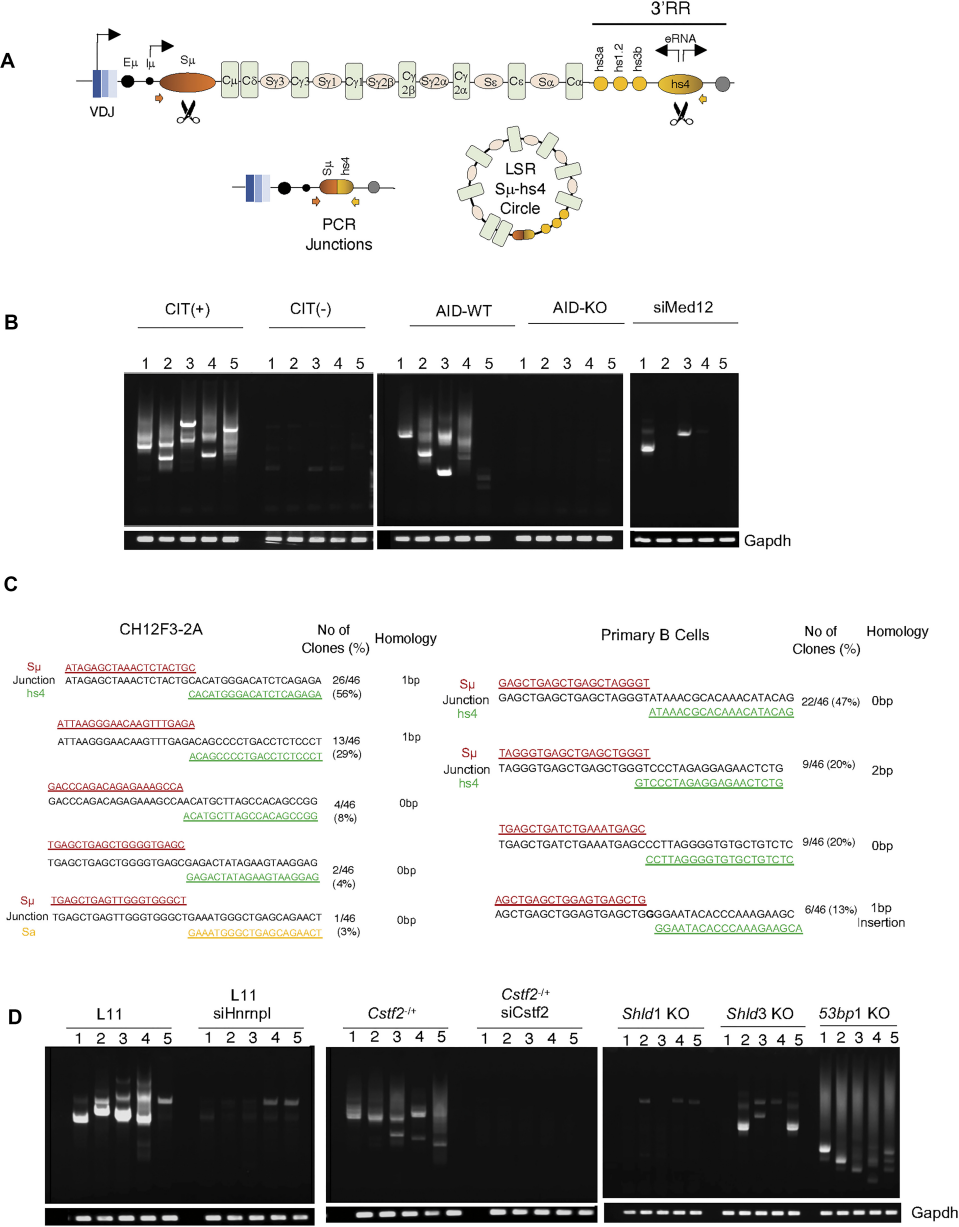
Depletion of hnRNPL and CstF64 disrupted LSR. (**A**) A schematic illustration of the IgH locus highlighting the S regions and the DNase hypersensitive (hs) sites at the 3′RR. During LSR, the DSB ends at Sμ, and hs4 randomly joins, excising the large intervening region as an excised DNA loop. The scissors indicate AID-induced DSBs on specific sites that are associated with LSR and are analyzed in this study. The bidirectional arrows over the hs4 region indicate the sense-antisense hs4-eRNA transcription from there. The thicker colored arrows indicate the position of forward and reverse primers used to perform LSR in CH12F3-2A cells. (**B**) Gel electrophoresis of LSR-PCR in CH12F3-2A cells and primary B cells after CSR stimulation. No LSR-PCR signal was detected without CSR stimulation [CIT (−)] or in stimulated AID-KO B cells. The reduced LSR-PCR signal (last panel) indicates impaired AID-induced DSB following Med12 knockdown in CH12F3-2A cells. The LSR-PCR was performed using gDNA isolated from cells stimulated for IgA and IgG1 switching in CH12F3-A and primary B cells, respectively. Each lane represents an independent PCR replicate, labeled 1–5, for each set. The PCR of the *Gapdh* locus acted as a loading control. (**C**) Confirmation of the joining of Sμ (red) and hs4 (green) regions in CH12F3-2A and primary splenic B cells. Some clones also showed Sμ–Sα joining, indicated in yellow. The LSR-PCR bands (B) from each lane were excised for cloning and sequencing. (**D**) Gel electrophoresis displaying LSR-PCR results from CH12F3-2A cells with hnRNPL and CstF64 knockdown, along with the KO of DNA repair factors (53BP1, Shieldin complex proteins).

Given that our previous study indicated that hnRNPL might be involved in the DNA repair phase of CSR [18], the presence of hnRNPL–CstF64 in the S and 3′RR regions led us to investigate whether it also affects LSR (Fig. 8A), as LSR involves recombination between the S region and 3′RR, unlike CSR, which entails S–S recombination [20].

Since there was no prior report of LSR detection in CH12F3-2A cells, we initially tested the assay by comparing CIT-stimulated and unstimulated cells, as CIT induces AID expression, which is essential for LSR. We also used primary B cells stimulated with LPS and IL-4 from *Aicda^+/+^* and *Aicda^−/−^* mice to assess the performance of our assay. We attempted to PCR amplify the recombination junctions between the Sμ and the hs4 3′RR using the previously described method for primary B cells [20]. LSR-derived PCR products were successfully detected from gDNA isolated from stimulated CH12F3-2A cells and *Aicda^+/+^* primary B cells but not from unstimulated CH12F3-2A and *Aicda^−/−^* cells (Fig. 8B), confirming that AID-induced DNA breaks occur at both S and 3′RR regions in CH12F3-2A cells. Consistently, reduced LSR product was also observed in CH12F3-2A cells deficient in MED12, which is required for 3′RR activation and AID-induced DNA breaks at S and 3′RR regions [10]. To further validate, we cloned LSR-derived PCR fragments from stimulated samples and sequenced them, with lengths ranging from 100 to 1000 bp, confirming the joining between Sμ and 3′RR in CH12F3-2A and primary B cells (Fig. 8C).

Next, we conducted LSR assays in *Hnrnpl^+/−^* and *Cstf2^+/−^* CH12F3-2A cells, which were transfected with siHnrnpl and siCstf2, respectively. After 24 h of siRNA transfection, the cells were treated with CIT for 48 h, followed by analysis of the gDNA. No LSR product was detected when hnRNPL or CstF64 was depleted (Fig. 8D, left and middle panels). Since CSR primarily occurs through C-NHEJ-mediated repair, we were curious whether LSR requires *bona fide* C-NHEJ components, such as 53BP1 or the Shieldin complex [37]. Analysis of gDNA derived from *Shld1*^−/−^, *Shld3*^−/−,^ or *53bp1*^−/−^ CH12F3-2A cells did not show any significant LSR (Fig. 8D, right panel), suggesting that, similar to CSR, LSR also may require the NHEJ complex [37–39]. Consistently, LSR junctions obtained were mainly blunt or 1–2 bp microhomology, which falls within the NHEJ category (Fig. 8C). The background signal detected in the *53bp1^−/−^* sample (Fig. 8D, right panel) is likely due to the residual recombination efficiency in these cells compared to the Shieldin deficiencies.

### hnRNPL and CstF64 promote NHEJ-mediated repair during CSR

**Figure 9.**
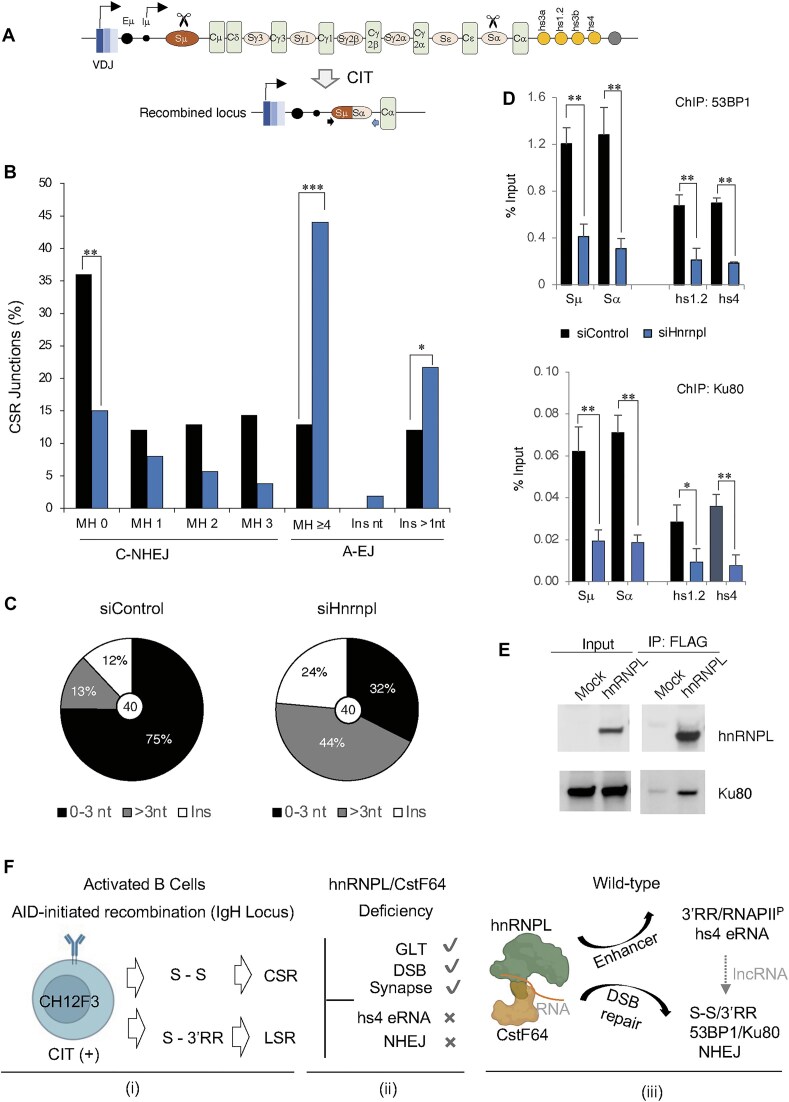
hnRNPL supports NHEJ-mediated repair by facilitating 53BP1 and Ku80 recruitment. (**A**) Schematic representation of the mouse IgH locus before and after CSR to IgA following CIT stimulation. Arrows indicate the positions of primers used to amplify Sμ–Sα junctions for CSR junction analysis. (**B**) Quantification of CSR junctions from gDNA isolated from CIT-stimulated CH12F3-2A cells transfected with siControl or siHnrnpl. The efficiency of Sμ–Sα junction formation was normalized to the siControl sample (set to 100%). MH indicates microhomology; blunt-end junctions are denoted as MH0. Statistical analysis was performed using Fisher’s exact test (**P ≤ .01; ***P ≤ .001). (**C**) Pie charts showing the proportion of clones repaired via classical NHEJ (C-NHEJ, MH 0–3 nt) versus alternative end-joining (A-EJ, MH > 3 nt and insertions [Ins]). The total number of unique junctions analyzed per condition is indicated in the center of each chart. (**D**) ChIP-qPCR showing the occupancy of 53BP1 and Ku80 at the recombining S regions and 3′RR of the IgH locus, before and after hnRNPL knockdown. The data represent three independent experiments. Statistical analysis was performed using an unpaired two-tailed *t*-test (*n* = 3; mean ± SD; *P ≤ .05, **P ≤ .01). (**E**) Co-IP analysis showing the interaction between hnRNPL and Ku80. Flag-tagged hnRNPL was expressed in HEK293T cells, followed by anti-Flag immunoprecipitation and western blotting with the indicated antibodies. (**F**) Proposed functions of hnRNPL in CSR and LSR. (i) DSB formation and recombination outcomes in activated CH12F3 cells. Upon CIT stimulation, AID introduces DSBs in the IgH locus, predominantly at S regions and, to a lesser extent, at the 3′RR. While S–S recombination events lead to productive CSR, recombination between S regions and the 3′RR can trigger LSR. (ii) Selective disruption of CSR-associated steps upon hnRNPL/CstF64 depletion, summarizing previously reported and newly observed molecular events. Unperturbed steps—such as GLT, DSB formation, and synapses—are indicated by check marks and are known to proceed normally. Impaired processes, including hs4 enhancer eRNA transcription and NHEJ-mediated repair, are marked with crosses (this study). (iii) CSR regulatory role of the hnRNPL/CstF64 complex. The scheme highlights two distinct axes: one showing their interaction with the hs4 enhancer at 3′RR, promoting RNAPII S2P and hs4-eRNA transcription. The other axis involves its interaction with the core NHEJ complex, including 53BP1 and Ku80, facilitating their recruitment and the repair of DSBs. The association of hnRNPL with eRNAs and lncRNAs, along with its RNA-sensitive chromatin binding, supports a model in which RNA molecules scaffold or modulate the repair process at the IgH locus.

Although we previously demonstrated that hnRNPL is dispensable for AID-induced DNA cleavage and likely functions downstream during the repair phase of CSR [18], its involvement in DSB end-joining had not been formally assessed. To investigate this, we analyzed recombination junctions in CH12F3-2A cells after siRNA-mediated knockdown of *Hnrnpl* and stimulation for IgM-to-IgA switching (Fig. 9A).

Sequencing of the Sμ–Sα junctions revealed a marked shift in the repair signature in hnRNPL-depleted cells. In WT cells, 75% of CSR junctions exhibited either blunt ends (0 nt microhomology [MH]) or short MH (≤3 nt), a pattern characteristic of C-NHEJ. By contrast, in hnRNPL-deficient cells, this proportion dropped to 32%, accompanied by a notable increase in longer MH usage (≥4 nt; 44%) and insertions (>1 nt; 24%), compared to 13% and 12%, respectively, in WT (Fig. 9B and C). The knockdown of *Cstf2* (CstF64) produced a similar defect in end-joining, with microhomology-mediated events (≥4 nt MH) increasing to 53%, possibly due to fewer insertion events than in hnRNPL knockdown (Supplementary Fig. S4A and B).

To directly test whether hnRNPL and CstF64 are required for C-NHEJ, we employed a GFP-based end-joining reporter system [26], where two I-*Sce*I sites flank a stop cassette (Thymidine Kinase; TK) between the CMV promoter and EGFP (Supplementary Fig. S5A). Upon I-*Sce*I-induced DSBs, efficient C-NHEJ results in loss of the intervening cassette and restoration of GFP expression. Depletion of hnRNPL or CSTF64 significantly reduced the frequency of GFP-positive cells, indicating impaired C-NHEJ repair (Supplementary Fig. S5B). This reduction was corroborated by gDNA analysis, which showed a consistently lower yield of recombined products across independent experiments (Supplementary Fig. S5C). The depletion of hnRNPL or CstF64 has no impact on ectopically expressed GFP (Supplementary Fig. S5D). This observation further validates that the GFP-positive cells are produced exclusively through NHEJ (Supplementary Fig. S5A), which was compromised in the absence of hnRNPL or CstF64. These results are in agreement with a previous finding using similar reporter systems for hnRNPL in colorectal cancer cells [24]. Collectively, these data indicate that both hnRNPL and CstF64 are critical for efficient C-NHEJ, not only during CSR but also potentially in maintaining general genome stability.

To probe the mechanism underlying this defect, we investigated whether hnRNPL influences the recruitment of core C-NHEJ factors to switch regions. Since hnRNPL proteomics identified an interaction with 53BP1, we first examined 53BP1 recruitment to the IgH locus. ChIP-qPCR following hnRNPL knockdown revealed a significant reduction in 53BP1 occupancy at the Sμ and Sα switch regions, as well as the 3′RR sites (Fig. 9D). Strikingly, Ku80—a key DNA DSB end-binding protein—was also diminished at both the S regions and the 3′RR. Co-IP confirmed that hnRNPL and CstF64 interact with Ku80; however, CstF64 exhibited a weaker interaction with Ku80 (not shown), indicating a predominant interaction with hnRNPL (Fig. 9E). Together, these data suggest that hnRNPL knockdown disrupts recruitment of essential C-NHEJ factors at the S regions and 3′RR, thereby impairing the formation of a functional repair complex. This mechanistically explains the pronounced defects in both CSR and LSR observed upon hnRNPL/CstF64 depletion in CH12F3-2A cells.

### The reduced recruitment of 53BP1 and Ku80 is not caused by decreased transcription

Since hnRNPL is known to contribute to gene expression regulation [40–42], we wondered whether the impaired recruitment of 53BP1 and Ku80 observed in the S and 3′RR regions was due to a decrease in the transcription of these genes in CH12F3-2A cells. Therefore, we selected a set of DNA repair and epigenomic regulatory genes recently found to be affected by hnRNPL in primary B cells: *Atm*, *Shld2*, *53bp1*, *Xrcc5/Ku80*, *Kdm6a*, and *Sirt1*. To confirm that the full-length gene or the entire CDS is sufficiently expressed, we amplified the CDS regions of these genes from siControl- and siHnrnpl-treated cells. The complete CDS of all the genes was successfully amplified from both conditions and yielded more or less comparable results with the control (Supplementary Fig. S6A). Expression of AID, which was not reported to change in primary B cells in hnRNPL deficiency [41], was monitored in parallel. Quantitative evaluation by RT-qPCR further confirmed no significant difference in DNA repair gene transcription after hnRNPL knockdown, which also aligned with 53BP1 and Ku80 expression at the protein level (Supplementary Fig. S6B and C).

In the case of *Kdm6a* and *Sirt1*, we did not detect any changes in *Kdm6a* expression but observed a reduction in *Sirt1* expression, as reported for the CH12F3-2A cells [41] (Supplementary Fig. S6D). We did not detect any notable changes in H3K27me3 and H3K9 acetylated histones’ posttranslational modification by ChIP analysis on the S or 3′RR (Supplementary Fig. S4C). Since the elevation of many long noncoding RNAs is a marked feature of hnRNPL deficiency [41], we examined lncRNA-Neat1, which sharply contrasts with the repair genes or *Sirt1* tested, showing significantly higher expression. Since Neat1.2 (short isoform) plays a critical role in promoting 53BP1-mediated NHEJ and genomic stability [43], an increase in Neat1.2 (long isoform) that does not support NHEJ may have a negative impact (Supplementary Fig. S6D). We confirmed the depletion of hnRNPL and CSR impairment, but no change in cell proliferation and viability (Supplementary Fig. S6E).

We also confirmed in the NHEJ reporter cell line that neither hnRNPL nor CstF64 depletion affects the transcription or protein expression levels of 53BP1 and Ku80 (Supplementary Fig. S7A–D). Similarly, the pCMV promoter-driven transcription of the TK gene from the integrated reporter cassette also showed no reduction in transcription. Consistent with this observation, we observed earlier that the ectopic expression of GFP remains unaffected upon depleting hnRNPL or CstF64, excluding any transcriptional disruption (Supplementary Fig. S5D). The cell cycle profile also showed comparable results between the control and hnRNPL or CstF64 knockdown (Supplementary Fig. S7F).

The recent study also suggests that primary B cells undergo apoptosis and proliferation arrest in the absence of hnRNPL, which can be directly linked to the transcriptional deregulation observed [41]. We thoroughly examined cell proliferation in CH12F3-2A cells using CFSE labeling, which did not indicate any significant impairment in cell proliferation in hnRNPL-knockdown cells (Supplementary Fig. S8). The CstF64-deficient CH12F3-2A cells produced similar results in cell proliferation using the same assay as WT (Supplementary Fig. S9). It is likely that CH12F3-2A cells, especially with the high expression of *Bcl2* (Supplementary Fig. S6E), demonstrate improved performance in the transient gene knockdown system compared to primary B cells.

## Discussion

### The vRRMs of hnRNPL are crucial for promoting CSR

The current study uncovers the mechanism of IgH super-enhancer transcription and hs4-eRNA regulation through association with hnRNPL and CstF64 protein complex. The fine mapping of hnRNPL RNA binding domain reveals that unconventional β sheets uniquely present at vRRM2 and vRRM3 equally contribute to the CSR. The hnRNPL consists of four RRMs and is known to bind CA repeat-rich RNA via an RNA looping mechanism in which each domain is responsible for binding RNA to various degrees [29, 44]. In the context of CSR, we found that vRRM2 and vRRM3 are the primary contributors, whereas RRM1 is partially responsible, likely by facilitating the RNA binding to vRRM2 and vRRM3.

The hnRNPL-RIP in activated B cells pulled down eRNAs from hs4-3′RR, and the eRNA-hnRNPL binding was found to be dependent on the vRRM motifs. A similar eRNA-hnRNPL interaction was previously reported for the target gene locus of myoblast differentiation, suggesting hnRNPL’s intimate relation with the transcriptional hub [42]. Additionally, proteomics analysis of protein bound to hnRNPL vRRMs revealed that polyadenylation factor CstF64 is one of the major interacting partners. The depletion of either hnRNPL or CstF64 affects hs4-eRNA transcription, CSR, and LSR. However, the depletion of hnRNPL did not influence SHM, AID-induced DSB, or S–S synapses, leaving its CSR-specific role at the post-break DNA repair phase [18]. Consequently, NHEJ factor 53BP1 dissociates from hnRNPL in the absence of vRRMs, and the deficiency of either leads to NHEJ defect, suggesting the repair/recombination-promoting function of the hnRNPL/CstF64 complex.

### The hs4-3′RR enhancer is regulated by the hnRNPL/CstF64 complex

Enhancer-promoter activation is an initial step in the activation of any target gene, requiring a specific set of protein complexes, including mediators, RNAPII machinery, coactivators, and others [33]. Similarly, IgH 3′RR enhancers require MED12 for enhancer activation by recruiting p300 and deposition of activation histone mark, H3K27ac [10]. The depletion of hnRNPL or CstF64 in activated B cells reduced enhancer transcription without affecting switch germline transcription, suggesting its function in 3′RR-specific transcriptional regulation. Interestingly, we observed that hs4-eRNA transcripts do not necessarily require polyadenylation, as a large percentage of them are non-polyadenylated. Consistently, the depletion of two essential subunits of the polyadenylation complex, required for polyadenylation [31], did not affect hs4-eRNA transcription or CSR. Similarly, the ineffectiveness of pan polyA inhibitor cordycepin [32] on hs4-eRNA transcription was also evident. Thus, we provide multiple lines of evidence that the polyadenylation factor CstF64 plays a non-canonical function in CSR.

Since eRNA polyadenylation was not the underlying cause of CSR defect, we examined the transcriptional machinery for eRNA synthesis because the direct interaction of hnRNPL and CstF64 with RNAPII has been reported previously [36]. In general, RNAPII promoter-proximal pausing is a well-known mechanism for controlling the primary transcription, which includes initiation, elongation, and termination [45]. Following pre-initiation complex formation, RNAPII frequently pauses synthesizing nascent RNA of 20–60 nt. The early elongation and productive elongation signals are initiated by RNAPII phosphorylation at C-terminal repeated sequences (YSPTSPS), such as S5P at early elongation, whereas S2P at productive elongation [45, 46]. Indeed, our data show that CH12F3-2A cells undergoing CSR have a high level of RNAPII-S2P in the switch and enhancer region, which also correlates with similar enrichment profiles of CstF64. Remarkably, the depletion of hnRNPL or CstF64 drastically reduced the S2P form of RNAPII, indicating a direct involvement of the hnRNPL–CstF64 complex with the productive elongation.

The positive transcription elongation factor b (P-TEFb), composed of CDK9 and cyclin T1, facilitates transcriptional elongation by phosphorylating serine 2 of the RNAPII CTD, the DSIF subunit Spt5, and the NELF complex [47]. Phosphorylation of NELF triggers its dissociation from the paused RNAPII complex, while phosphorylation of DSIF converts it into a positive elongation factor that remains associated with actively transcribing RNAPII [45, 47]. We found that transcription at the IgH hs4 3′RR enhancer was further reduced upon treatment with the P-TEFb kinase inhibitor flavopiridol, indicating that RNAPII phosphorylation is essential for eRNA production at this regulatory region (Supplementary Fig. S2E). This is consistent with prior findings showing that Spt5 deficiency diminishes transcriptional elongation at the IgH 3′RR without altering paused RNAPII occupancy, leading to impaired CSR [9].

Enhancer-derived RNAs produced from activated enhancers are known to support multiple regulatory functions, including the release of promoter-proximal RNAPII pausing [48]. In particular, eRNAs can bind the RNA recognition motif of NELF-E, facilitating its dissociation from the pausing complex and enabling the transition to productive elongation. Consistent with this, we found that depletion of hs4-eRNAs either by ASOs or by CRISPRi using dCas9-KRAB targeting hs4 led to reduced levels of RNAPII S2P at both the 3′RR and S regions (Fig. 7), suggesting that hs4-eRNAs act in an autoregulatory manner to promote elongation. We further observed that hs4-eRNAs facilitate the recruitment of hnRNPL, CstF64, and likely P-TEFb to the enhancer, supporting a model in which the hnRNPL–CstF64–eRNA complex promotes transcriptional elongation at the hs4 enhancer during CSR.

### Elusive role of 3RR-derived eRNA in CSR: hs4-eRNA dependency of CH12F3-2A cells

Although the precise function of eRNAs in CSR remains unresolved, evidence suggests that transcripts from individual hs sites within the 3′RR of the IgH locus modulate enhancer activity, chromatin architecture, and potentially AID recruitment to the IgH locus [9, 12, 13, 49]. The palindromic structure of the 3′RR, with hs1.2 flanked by hs3a and hs3b, has been shown to support cooperative enhancer function essential for CSR. Deletion of individual enhancer elements typically has mild effects, but combined deletions, such as of hs3b and hs4, drastically impair CSR and Ig expression [8, 11, 50]. It has also been reported that hs4 may be unnecessary for Ig isotype switching *in vivo*, as evidenced by the generation of hs4-deleted mice [51]. However, evaluating its effect on IgA switching in activated B cells is challenging due to the low efficiency of IgA switching, although secreted IgA levels exhibited a declining trend in this model.

In contrast, CH12F3-2A cells, which undergo IgA switching with high efficiency, show an unusual sensitivity to the perturbation of hs4-derived eRNA. In particular, the depletion of components like MED12 leads to a drastic reduction in hs4-eRNA, enhancer deactivation, impaired AID recruitment, and CSR [10]. Further insights into hs4 function and eRNA regulation have emerged from comparisons between WT and engineered NCΔ CH12F3 cells (Supplementary Fig. S10A and B) [52, 53]. Notably, the basal hs4 transcription levels are significantly higher in NCΔ than in WT CH12F3-2A cells, and this difference is further amplified in the NCΔ derivative, in which a large region encompassing the Sα and 3′RR on the non-productive IgH allele is deleted (Supplementary Fig. S10C). Transcription from hs1.2 sites is very low and less responsive to CIT induction in WT CH12F3-2A cells, but not NCΔ cells, which show higher hs1.2 transcription and responsiveness to CIT (Supplementary Fig. S10D). This phenomenon in NCΔ cells has been described as having “constitutively active” Sα and 3′RR regions, with elevated eRNA expression [12, 53]. However, the study lacks a comparison with the parental WT CH12F3 line, which has been known since its establishment that the Sα region is constitutively active, as αGLT is produced at a very low level but is highly inducible by CIT [22]. We reproducibly observed the phenomenon in WT CH12F3-2A cells that exhibit low basal αGLT and hs4-eRNA but robust upregulation of those upon CIT stimulation, resembling the inducible pattern seen in primary B cells [22, 53]. Importantly, NCΔ cells also show several-fold stronger induction response to CIT (Supplementary Fig. S10C–E), indicating that these enhancers remain stimulus-responsive rather than being fully constitutive, unlike the truly constitutive nature of μGLT transcription (Supplementary Fig. S10F). The elevated basal eRNA levels observed in NCΔ cells may instead reflect *cis*-derepression or the disruption of inter-allelic regulatory communication (transvection), whereby the intact 3′RR on one allele modulates transcription or AID recruitment on the other [54]. Interestingly, however, AID induction was higher in WT cells, even though CSR efficiency remained similar, indicating that AID levels in NCΔ cells are sufficient (Supplementary Fig. S10G and H).

A genomic deletion of hs4 in the WT and NCΔ CH12F3 background may provide a more direct assessment of hs4 function and CSR in these highly responsive and inducible models. Additionally, it remains to be tested whether hs4-deficient primary B cells respond differently under more optimized cytokine conditions, such as with APRIL, retinoic acid, and BAFF [55].

Depletion of hnRNPL in CH12F3-2A cells leads to reduced hs4-derived eRNA expression, likely due to impaired RNAPII elongation. Unlike MED12 deficiency [10], however, this reduction does not affect S-region DNA breaks or S–S synapsis, but specifically impairs NHEJ and CSR. Here, we show that the hnRNPL/CstF64 complex binds hs4-derived eRNAs and interacts with key NHEJ proteins, including 53BP1 and Ku80. Whether these NHEJ factors directly associate with hs4-eRNAs in activated B cells remains to be determined.

Nevertheless, our findings suggest the possibility of an RNA-mediated layer of CSR regulation, potentially involving hnRNPL, which is known to interact with chromatin-associated long noncoding RNAs (lncRNAs). Both 53BP1 and Ku80 have also been shown to bind various noncoding RNAs that modulate their DNA repair functions. Yet, the role of noncoding RNAs—including eRNAs—in CSR-associated NHEJ remains largely unexplored [56, 57]. Our data support the hypothesis that hs4-derived eRNAs, together with hnRNPL and possibly other lncRNAs, may help stabilize or scaffold DNA repair factors, functioning analogously to damage-induced or structural RNAs during CSR (Fig. 9F).

### hnRNPL–CstF64 complex contributes to NHEJ-mediated DNA repair

In CSR, AID induces DSBs in the universal donor S region (Sμ) and one of the downstream acceptor S regions (Sx), leading to an intrachromosomal deletional recombination, which unites the DSB-ends between Sμ and Sx through NHEJ-mediated repair [39]. Like switch repeat sequences that are the frequent target of AID-induced DSBs [58], the 3′RR sequence also contains numerous switch-like repeats resembling S-regions and is thus also subjected to AID-induced DSBs. Therefore, recombination between Sμ-DSB and 3′RR-DSB can delete nearly the entire IgH locus, resulting in no Ig production, and is speculated to cause cell death due to BCR loss; hence, it is referred to as LSR [20]. Therefore, LSR not only eliminates BCR-negative cells but also protects the IgH locus from undesired recombination with other genomic loci.

Classical-NHEJ is CSR’s primary DSB repair pathway, with key cofactors such as 53BP1, Shieldin, and Ku70/Ku80 playing essential roles [37–39]. Consistent with this, the loss of LSR observed in hnRNPL-, CstF64-, 53BP1-, Shieldin-, or Med12-deficient CH12F3-2A cells supports the notion that the 3′RR, like S regions, is mainly repaired through NHEJ. Analysis of Sμ-3′RR recombination junctions revealed blunt ends or short (1–2 bp) microhomologies—hallmarks of NHEJ. A minority of junctions were more complex, mirroring prior observations in primary B cells [20]. Although not tested in our study, poly [ADP-ribose] polymerase 1 (PARP-1) has also been detected at the 3′RR during LSR, supporting the role of NHEJ and alternative end-joining (A-EJ) in this process [59]. Our ChIP analyses show that hnRNPL and CstF64 are more enriched at the Sμ and hs4 regions than at Sα, aligning with previous findings that hnRNPL preferentially binds Sμ over Sγ in activated primary B cells [41]. We also detected Ku80 Co-IP with hnRNPL and observed reduced Ku80 binding at the IgH locus upon hnRNPL depletion, implying that loss of hnRNPL may destabilize NHEJ factor recruitment more broadly (Fig. 9D).

Given that hnRNPL binding at the IgH locus is sensitive to RNase A treatment (Fig. 7H), we suspect that RNA molecules—such as eRNAs from the hs4 region of the 3′RR or other noncoding RNAs—facilitate its recruitment. The interaction between hnRNPL and 53BP1, as well as the association of NHEJ proteins with damage-induced noncoding RNAs, appears to be RNA-dependent [60, 61], suggesting that hnRNPL may contribute to CSR and LSR through RNA-guided assembly of DNA repair complexes. This may involve mechanisms such as liquid–liquid phase separation [56, 57], which has been implicated in the spatial organization of repair foci during CSR and potentially in LSR. In parallel, transcriptome analyses of hnRNPL-deficient B cells point to a predominant role in RNA transcription [41], though global expression changes in such settings often make it difficult to pinpoint direct links to CSR. We approached hnRNPL’s function by dissecting the specific steps required for CSR—including germline transcription, DSB formation, synapsis, repair, and 3′RR activation—and revealed that hnRNPL is specifically required for the hs4-enhancer transcription and repair complex assembly. Together, our findings support at least two mechanistic roles for hnRNPL in CSR/LSR and underscore the need to explore further both RNA-mediated recruitment and potential splicing/transcription-dependent pathways.

While transcriptome analyses of hnRNPL-deficient B cells emphasize its role in RNA splicing, global changes in such datasets make it difficult to pinpoint direct links to CSR. In contrast, our focused analysis of key CSR stages revealed defects specifically in 3′RR transcription and DNA repair, but not in earlier steps such as germline transcription or synapsis. These findings support at least two modes of hnRNPL action—enhancer regulation and NHEJ facilitation—highlighting the need to further investigate both RNA-mediated and splicing/transcription-related mechanisms in antibody diversification. Since hnRNPL affects RNAPII phosphorylation and eRNA transcription at the IgH superenhancer, it could also impact other B cell enhancers and downstream gene expression—an interesting area for future research.

## Supplementary Material

gkaf810_Supplemental_Files

## Data Availability

All data needed to evaluate the conclusions in the paper are present in the paper and/or the supplementary materials. This study does not include any data deposited in external repositories.

## Notes

Present address: Laboratory for Mucosal Immunity, Centre for Integrative Medical Sciences, RIKEN Yokohama Institute, Yokohama, Kanagawa 230-0045, Japan
