# Supplementary material for: hnRNPL–CstF64 complex: coordinating CSR and LSR in IgH locus recombination dynamics through eRNA and NHEJ regulation
Source: Nucleic Acids Res. 2025 Sep 3;53(16):gkaf810. doi: 10.1093/nar/gkaf810 (PMC12407099; doi:10.1093/nar/gkaf810)
Supplement: gkaf810_Supplemental_Files [file gkaf810_supplemental_files.zip › Supplementary Figures (R2).pdf]

Supplemental Information

hnRNPL-CstF64 Complex: coordinating CSR and LSR in IgH locus recombination dynamics through eRNA and NHEJ regulation

Farazul Haque[1,#], Mikiyo Nakata[1] Hidetaka Kosako[2], Tasuku Honjo[1*], Nasim A. Begum[1]

[1]Department of Immunology and Genomic Medicine, Centre for Cancer Immunotherapy and Immunobiology, Kyoto University Graduate School of Medicine, Kyoto 606-8501, Japan

[2]Division of Cell Signaling, Institute of Advanced Medical Sciences, Tokushima University, Tokushima 770-8503, Japan

[#] Current address: Laboratory for Mucosal Immunity, Centre for Integrative Medical Sciences, RIKEN Yokohama Institute, Yokohama, Kanagawa, 230-0045 Japan

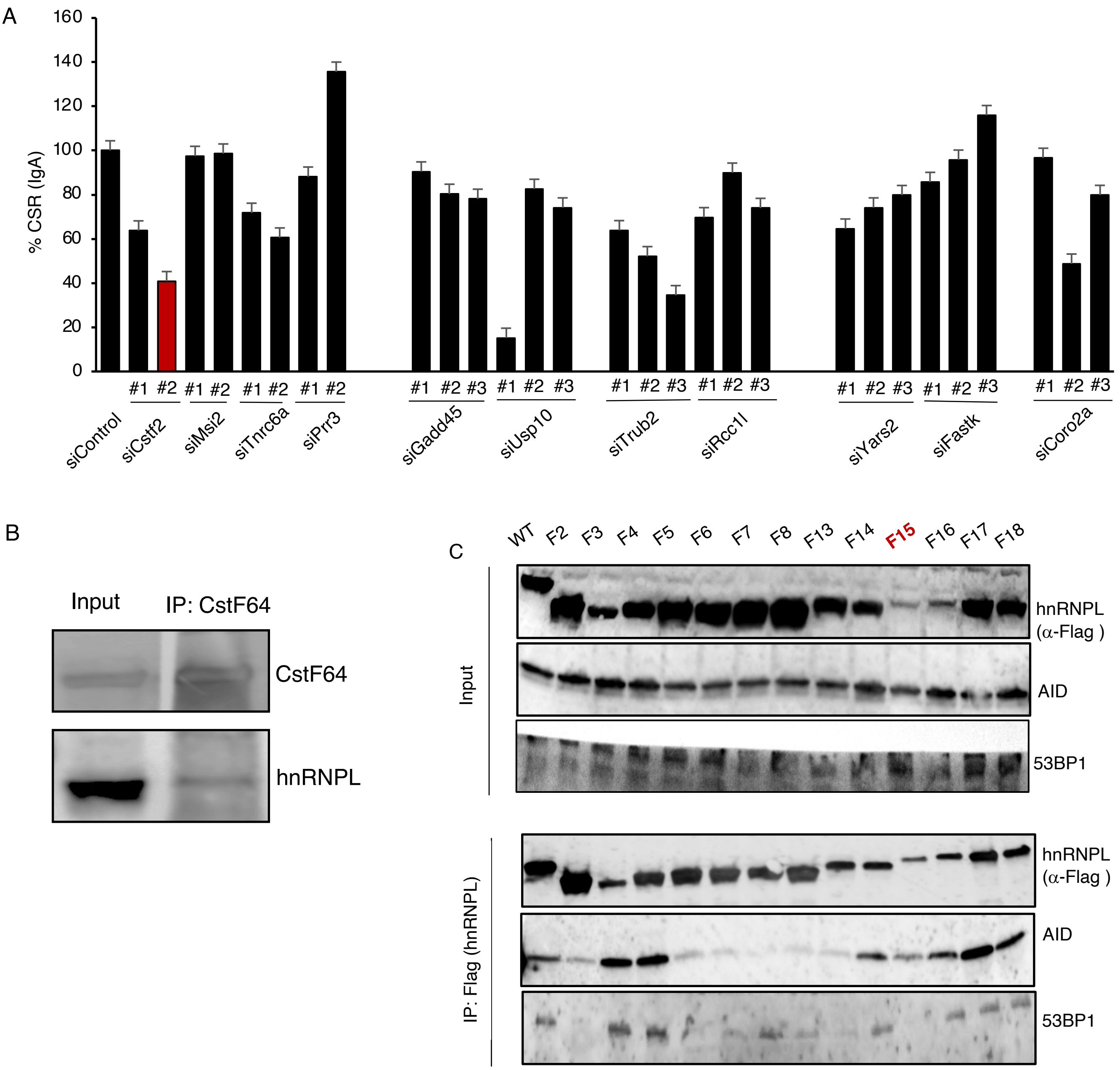

Supplementary Figure 1. Proteins that interact with hnRNPL WT but not with the F15 mutant

(A) CH12F3-2A cells were transfected with siRNAs targeting candidate genes identified as dissociated from the hnRNPL F15 mutant, followed by CSR stimulation with CIT for 24 and 48 hours. Each gene was targeted by two or three distinct siRNA oligos, as indicated by the "#" symbols below the bars. CSR efficiency was measured and expressed as a percentage relative to cells transfected with scrambled control siRNA (siControl). The red bar highlights the most promising candidate gene selected for further investigation. Data represent the mean ± SD of three independent experiments. Statistical significance was assessed using a two-tailed Student's t-test (ns, $P > 0.05$). **(B)** Co-immunoprecipitation of endogenous CstF64 demonstrating its interaction with hnRNPL in HEK293T cells. **(C)** Western blot showing the interaction between hnRNPL, 53BP1, and AID. HEK293T cells were transfected with Flag-tagged wild-type hnRNPL (WT) and its mutant constructs, denoted by "F#" as in Figure 2. Flag immunoprecipitation (IP) was performed to detect associated proteins.

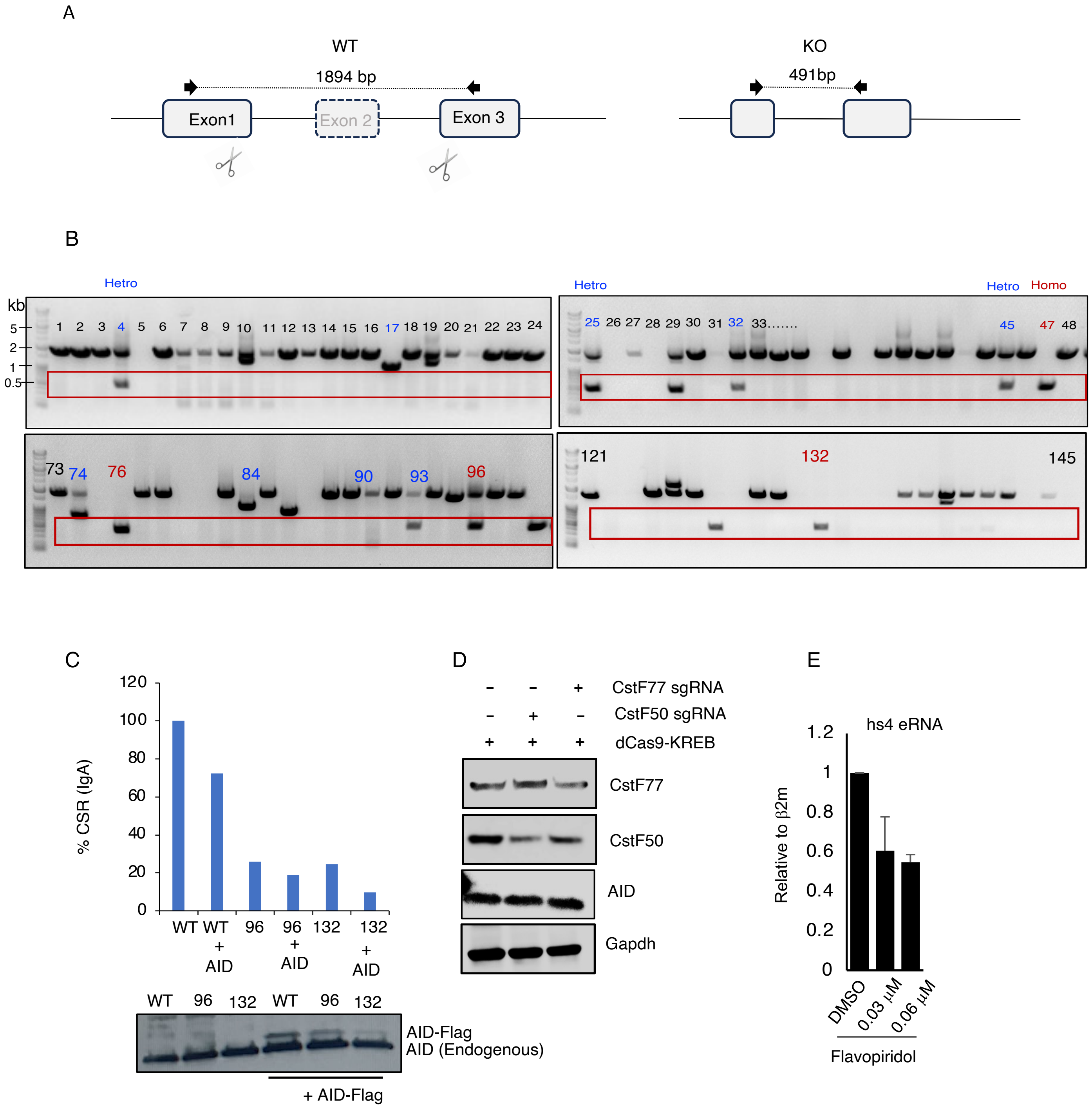

Supplementary Figure S2. Screening of *Cstf2* KO clones, CRISPR inhibition, and transcription inhibition.

(A) Schematic illustration of the CRISPR/Cas9 strategy targeting exon 1 and exon 3 of the mouse Cstf2 gene. CH12F3 cells were co-transfected with sgRNAs and Cas9, followed by single-cell cloning. **(B)** Agarose gel electrophoresis of genomic DNA PCR from individual clones showed the wild-type allele producing a 1894 bp band, while successful targeting produced a 491 bp band. For example, clone 25 was heterozygous, and clone 47 was homozygous KO, which were primarily used. **(C)** Analysis of additional homozygous *Cstf2* KO clones (96 and 132). All of which exhibited significantly reduced CSR efficiency regardless of ectopic AID expression. **(D)** Western blot analysis showing CRISPR interference (CRISPRi)-mediated knockdown of CstF50 and CstF77 subunits in CH12F3-2A cells. **(E)** Quantitative PCR analysis of hs4 eRNA expression in CH12F3-2A cells treated with flavopiridol, a transcriptional inhibitor. DMSO was used to dissolve the inhibitor and used as a control; DMSO concentration was the same as in 0.06 µM drug.

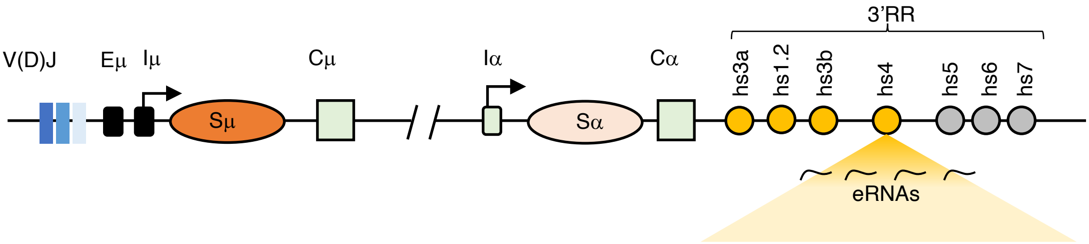


```
        1         10         20         30         40         50
        |         |          |          |          |          |
Pst I   CTGCAGACTC ACTGTTCACC ATGAACCCAG CTAGTCAGAT TCATATGTGA AACTCATATC 60
        AGCCTCTGCA CACACATACA CACACATACA CACATATTAC ACCCATGCAC ACACATGTAC
        ACATACATAC ACATGTACAC ATACATGTGT ACACACACAT ATAGAGAAGG CATTGGTGGG 180
        GAAAACATTA GGCCATGGCT ACAGTACAGG GCACAAGGAT GGTGGTACAG AATGAGGTCA
        GGCTGGGTCA GCATAACAAG AACACTTGGA CAAAGTGAGG GTAGTGTGTG TGTGTGTGTG 300
        TGTGTGTGTG TGTGTGTGTG CACGTTGAAA GTCTTCAGTA GACTGGTATC ACTAGCCCTG
        ATATGGGCAA CACAGCAAGC CTGGGTCACA CTCAAGCTGA GTATCAGGGT AGCCAGGGCC 420
        TTCTAACCAA GGGTAGATGC AGCCTGTGTT CCGTTTACTG ACCAGTGAGA AGCCATGAGC
        TGAACCAGAC CAGAAGACCC TTACTGTTCC CACCCCACCC CCACCCAGTT TAGTCTCAGC 540
        AAGACCCTGT ACTGTGGGCC ACAGCTCTCC TCTACACTCC ACCTGTAGCA CAAACACTAT
        TTGCAAACAT TTCTAAAAAG TAGTAGAACA GGAACCACAG AGCAGAGGGG GGGACTGGCG 660
        TGGAAAGCCC CATTCACCCA TGGGACTGAA ACTCAGGGAA CCAGAACCGT AAGGAGATTT
        GCATGGTGCT GGGGGAGGTT GGCCCTGGAT CAGTGAGCCC AGAGAGTTAC TGGTTTCTCA 780
        CTTCCATCAT GTCAACCTCC TCAACCCCCA AAAATGGCCA GGCCTAGGCT ATGGATGAGT
        TTCAATGACC AGGCCCTAAG GACGAGTCAC AGAGGACTTC CTGGTGGGCT CAGGCAGCAG 900
        ACCTGCTCAG ATGGATTGCA GAGCCAGAGG GAGCCATGGC CAGGAAGGCC AGACGCCTTA
        GGGGTGTGCT GTCTCTGCAT CCTTTGCCCT CTCTGCTCCT CCATCTGCCA TCTCACAATC 1020
        CCTGCTGTCG CTCTGGGGCC CAGACCTGGC CAGTCTGGGC ACAGTTACCT GTGGAATACA
        CCCAAAGAAG CAATCCCCAG CCTCAGGATC CACAACTACT TCCCCTACAG ACATGAGTGA 1140
        TCTCAGCCCA CATGTCTGGG GGCCACAGAA GCCCCTAAGA CCCTACTCTG CTAATAGGCC
        CTCCTCCCAC CAGCCAAGAC AATACACAGG CAAGGTGATG TGGATGAGAG TCACTCCATG 1260
        GGTACCTGTG TCTGAGATAC ACCCTGTGGG TATCCTGGCC AGAATCTGGT GACCAACCCA
        ACCTGTGTCC CTAGAGGAGT ACTCCGTGCC TGCACTCACC TACCCACCTA ACTCCAAGCT Hind III
        TGGTATGATG CAGAGCCCCT GTGTAGACCT CAAAGTCAGC CATAGGACAG GGTCAAGAAT
        GACTCTTCCT ACACATAAAG TCTTCTACTA AGACAGTAAG GTAGATACAC AAACATACCC 1500
        AAATGCAGAG ACACACAGGC ATGCAGAGAA GGCATGTAGA CACAAACGCA TGCATAAACG
        CACAAACATA CAGATATATG CTGACAAATA TACACAGCAA CTTACAAGTA CACAGACACA 1620
        CAAACAGACA AACATGCACA GACAGAAACA CACAGAGCTC AAAATCAAGT ATACACAGAC
        AAATTTACAC AGAGACTTAC AGATAGCTTA CACAGATATA TGAGACACAC CCAAACAGAC 1740
        ACACACATGG GGGCACAGAA AAACATACAA GCAGACACAT GCAGACTTAA AGACCCACAA
        GACATGGAGA GATACAAAAA CACACAACAC AGACACAGA                        1780
```

Supplementary Figure S3. **Mapping of RACE analyzed eRNAs from CH12F3-2A cells on the mouse IgH hs4-3' RR.**

The *PstI* and *HindIII* sites correspond to the 1381 bp (GeneBank Acc, S74166.1) region (Madison et al., Genes Dev 1994; 8, p2212). Numerous hs4-eRNAs of various lengths produced from the location are cloned by applying 5' and 3' RACE analyses. The transcriptional initiation (red arrowheads) and termination sites (green boxes) identified during analysis of the cloned transcripts are shown in the sequence. Since the directionality (sense/antisense) and full details of each transcript cannot be determined, the heterogeneous transcripts we mapped may also be considered "hs4-crossing transcripts."

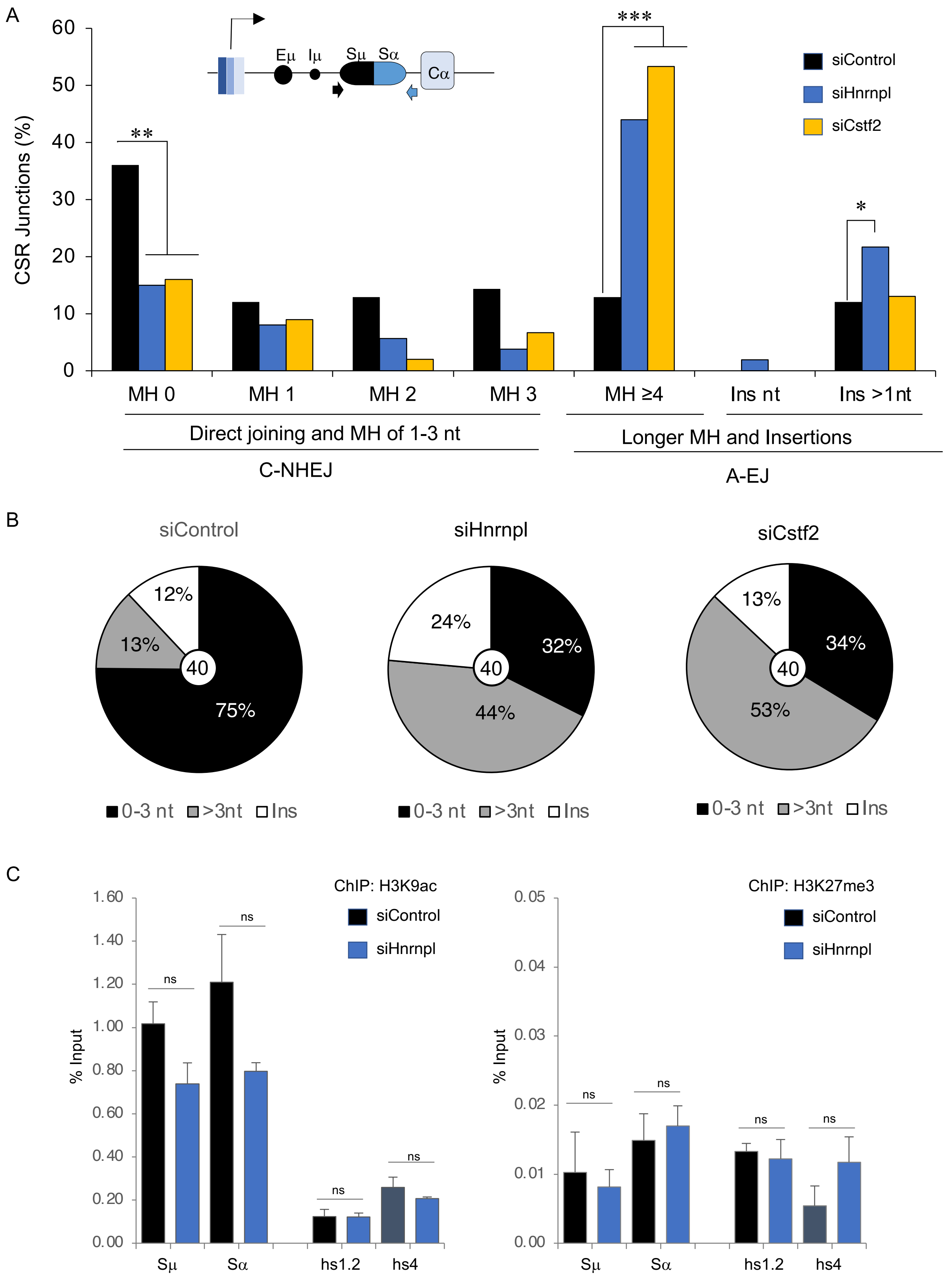

Supplementary Figure S4. Comparison of CSR junctions and histone epigenomic marks after hnRNPL or CstF64 depletion in CH12F3-2A cells.

(A-B) Knockdown of Hnrnpl (hnRNPL) or Cstf2 (CstF64) similarly impaired NHEJ-mediated repair of the Sμ and Sα recombination junctions. The siControl and siHnrnpl data are the same as in Figure 9B and C. **(C)** ChIP analysis, compiled from three experiments, shows no significant alteration in two histone modification marks at the IgH locus upon *Hnrnpl* knockdown in CH12F3-2A cells stimulated by CIT for 24 hours.

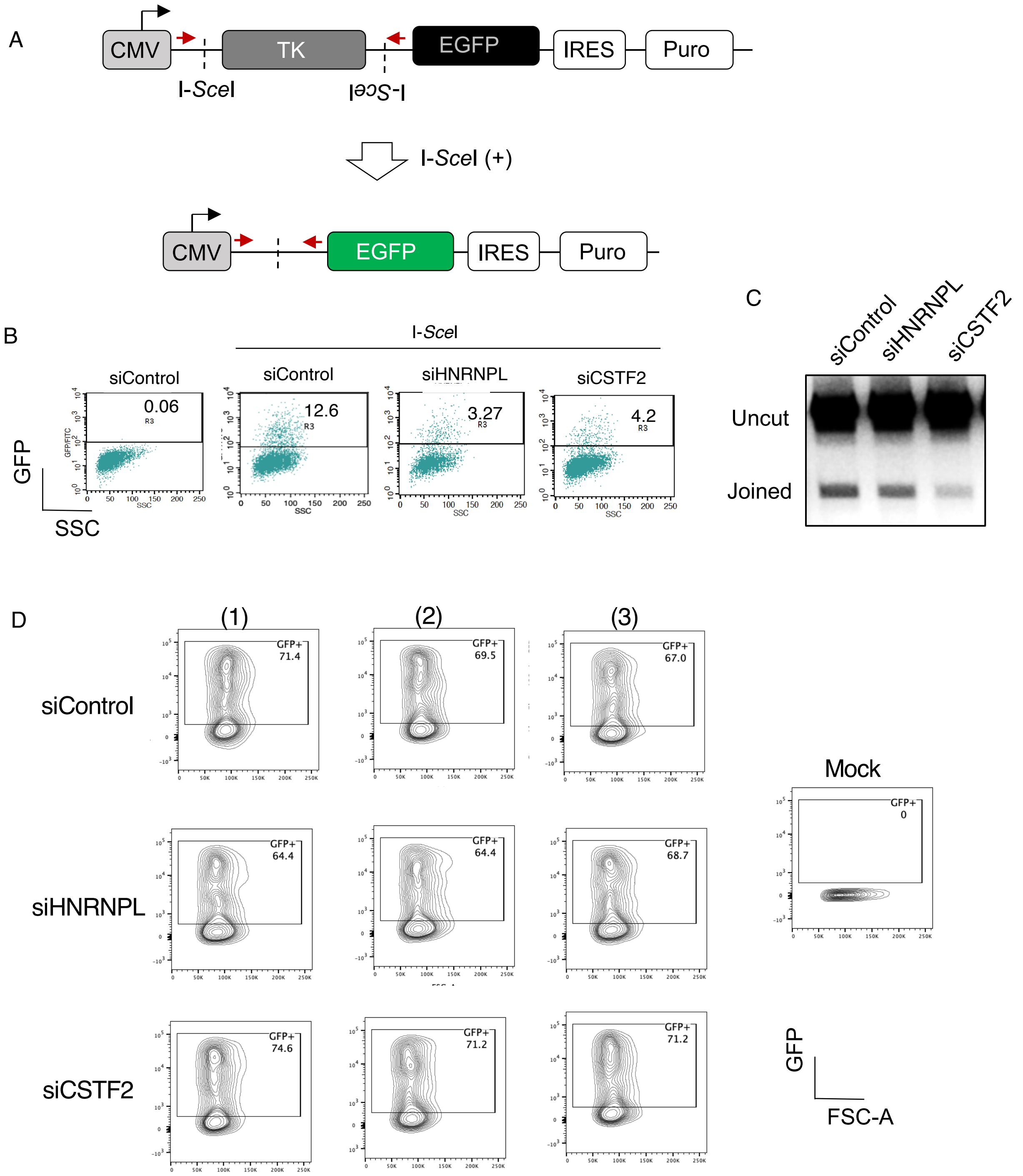

Supplementary Figure S5. The I-*Sce*I-induced DNA DSB repair by NHEJ in a GFP reporter assay.

(A) Schematic of the I-*Sce*I-based reporter system, illustrating the removal of TK and GFP expression following NHEJ. **(B)** The DSB repair by NHEJ was estimated by GFP expression. The cells were transfected in combination with the I-*Sce*I plasmid, either with siControl, siHNRNPL, or siHCSTF2. The GFP-positive cells were analyzed by FACS analysis after 48 h of transfection. The cell line does not express GFP in the absence of I-*Sce*I-induced DSB repair. **(C)** The DNA isolated from transfected cells was subjected to PCR to detect the successful repair by NHEJ. **(D)** Knockdown of HNRNPL or CSTF2 does not affect the expression of EGFP from the pCMV-EGFP vector. The reporter cell line was transfected in triplicate with pCMV-EGFP along with the indicated siRNAs, and without the I-*Sce*I. The % EGFP [GFP(+)] cells are displayed in the respective FACS plot.

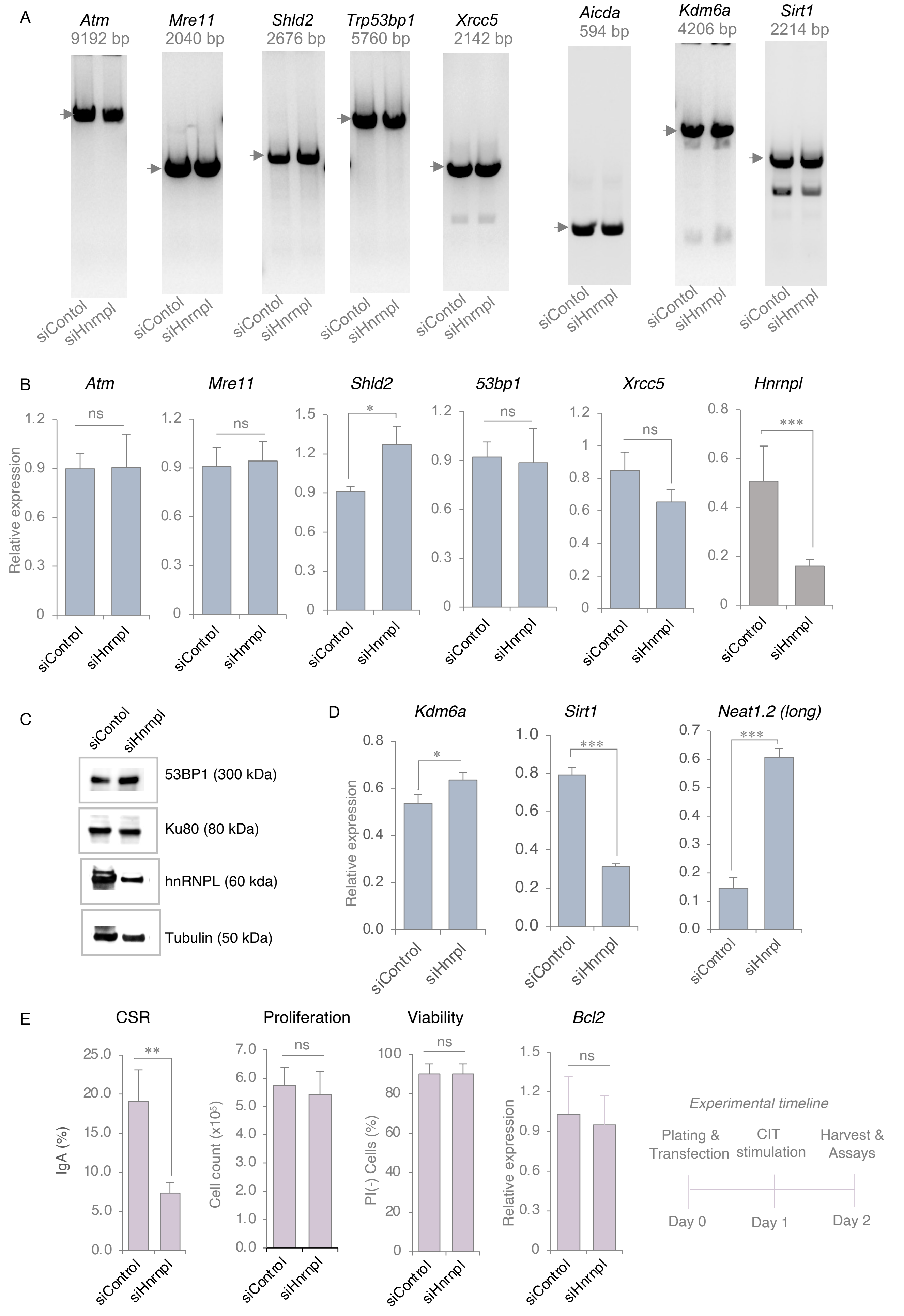

Supplementary Figure S6

Supplementary Figure S6. Expression of DNA repair genes remains unchanged upon hnRNPL depletion in CH12F3-2A cells.

(A) Representative agarose gel electrophoresis of RT-PCR products corresponding to the full-length coding sequences (CDS) of five DNA repair genes (*Atm*, *Mre11*, *Shld2*, *Trp53bp1*, and *Xrcc5*), two chromatin regulators (*Kdm6a* and *Sirt1*), and Aicda, a key regulator of CSR and SHM. Total RNA was isolated from CH12F3-2A cells transfected with siControl or siHnrnpl and stimulated with CIT for 24 hours. The expected CDS length for each gene is indicated below the gene name. Grey arrowheads denote the full-length CDS amplicons (See primers in the Supplementary Table 1).

(B) RT-qPCR analysis of the indicated DNA repair genes and Hnrnpl. Efficient hnRNPL knockdown confirms that transcript levels of the DNA repair genes remain unchanged upon depletion.

(C) Representative western blot validating hnRNPL depletion at the protein level. The expression of 53BP1 and Ku80 (encoded by *Trp53bp1* (*53bp1*) and *Xrcc5*, respectively) remained unchanged. consistent with transcript-level results in (A) and (B).

(D) RT-qPCR analysis of *Kdm6a*, *Sirt1*, and the long isoform of lncRNA Neat1 (variant 1.2). While *Kdm6a* levels were unaffected, *Sirt1* was moderately downregulated, and the lncRNA Neat1 (long form) was upregulated in response to hnRNPL knockdown (see Discussion).

(E) Summary of CSR, cell proliferation, and viability assays performed throughout the study. hnRNPL knockdown did not affect cell proliferation or viability. The right-most gel shows RT-PCR analysis of the anti-apoptotic *Bcl2* transgene, which was expressed at similar levels in control and hnRNPL-depleted cells. The experimental timeline used in these assays is also indicated.

(B, D, E) Statistical analysis was performed using an unpaired two-tailed t-test (n = 3; mean ± SD; $*P \leq 0.05$; $**P \leq 0.01$; $***P \leq 0.001$; ns, not significant).

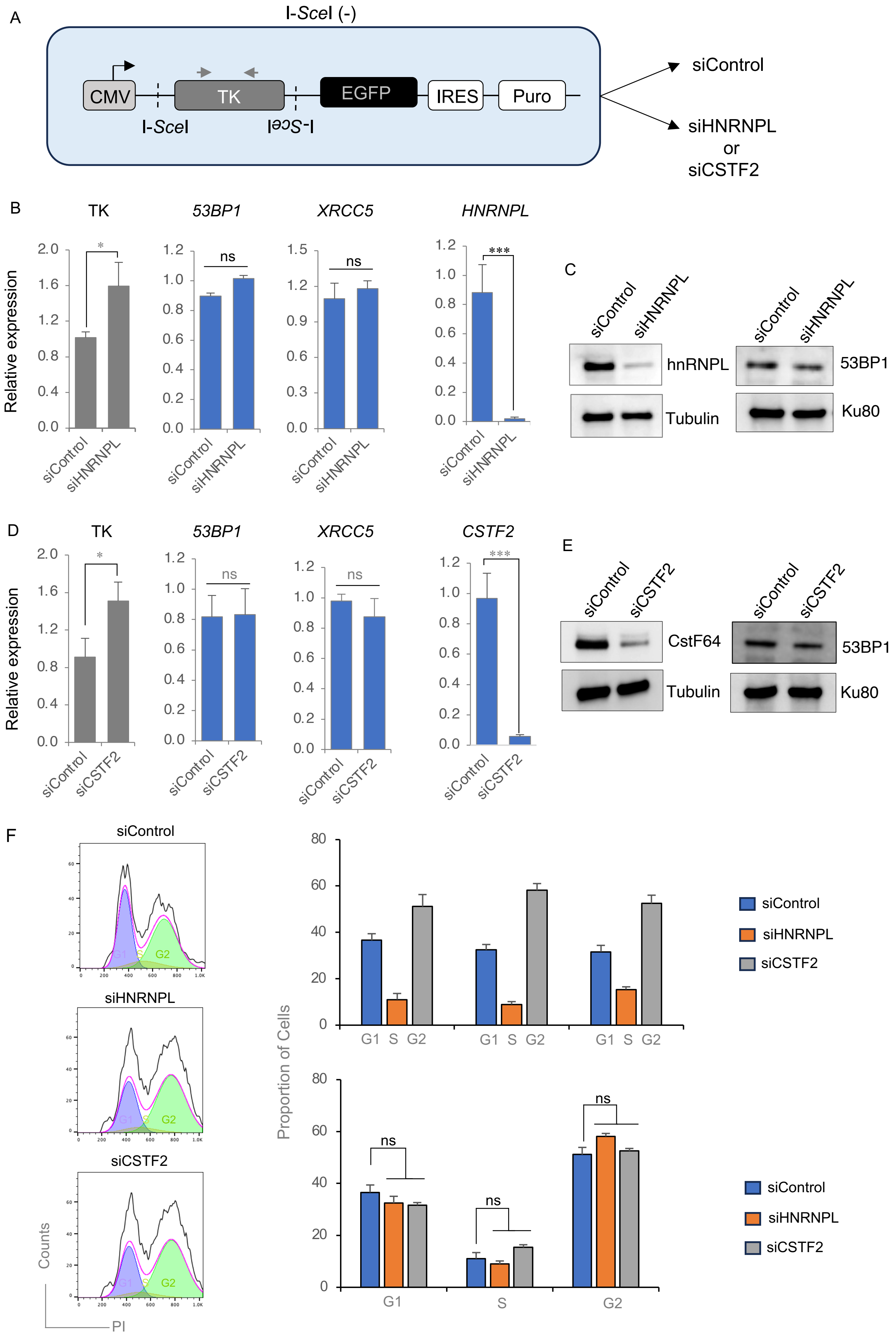

Supplementary Figure S7

Supplementary Figure S7. Knockdown of hnRNPL or CSTF2 does not alter expression of core NHEJ genes or cell cycle distribution.

(A) Schematic of the I-*Sce*I-based reporter construct used to monitor NHEJ-mediated DNA repair. The construct includes a CMV promoter driving the expression of thymidine kinase (TK), which is flanked by two I-S*ce*I sites that prevent downstream EGFP expression. An IRES-linked puromycin resistance gene (Puro) follows. The NHEJ reporter cell line was transfected with siRNAs targeting hnRNPL (siHNRNPL), CSTF2 (siCSTF2), or a non-targeting control (siControl).

(B) RT-qPCR analysis of TK, *53BP1*, *XRCC5*, and *HNRNPL* mRNA levels following hnRNPL knockdown. While HNRNPL transcription was significantly reduced, expression of TK and the NHEJ genes remained unchanged.

(C) Representative western blot showing efficient hnRNPL depletion.

(D) RT-qPCR analysis of TK, *53BP1*, and *XRCC5* following CSTF2 knockdown. While CSTF2 depletion did not significantly affect the expression of *53BP1* or *XRCC5*, a modest increase in TK transcript levels was observed.

(E) Representative western blot showing effective depletion of CstF64 (encoded by CSTF2).

(F) Cell cycle profiles determined by propidium iodide (PI) staining of nuclei and flow cytometry. Left: representative histograms showing G1, S, and G2 populations in siControl, siHNRNPL, and siCSTF2-treated cells. Right: quantification of the proportion of cells in each phase. No significant changes (ns) in G1, S, or G2 phase distribution were observed across treatments, indicating that hnRNPL or CSTF2 depletion does not impact cell cycle progression under these conditions.

(B, D, F) Statistical analysis was performed using an unpaired two-tailed t-test ($n = 3$; mean ± SD; $^{**}P \leq 0.01$; $^{***}P \leq 0.001$; ns, not significant).

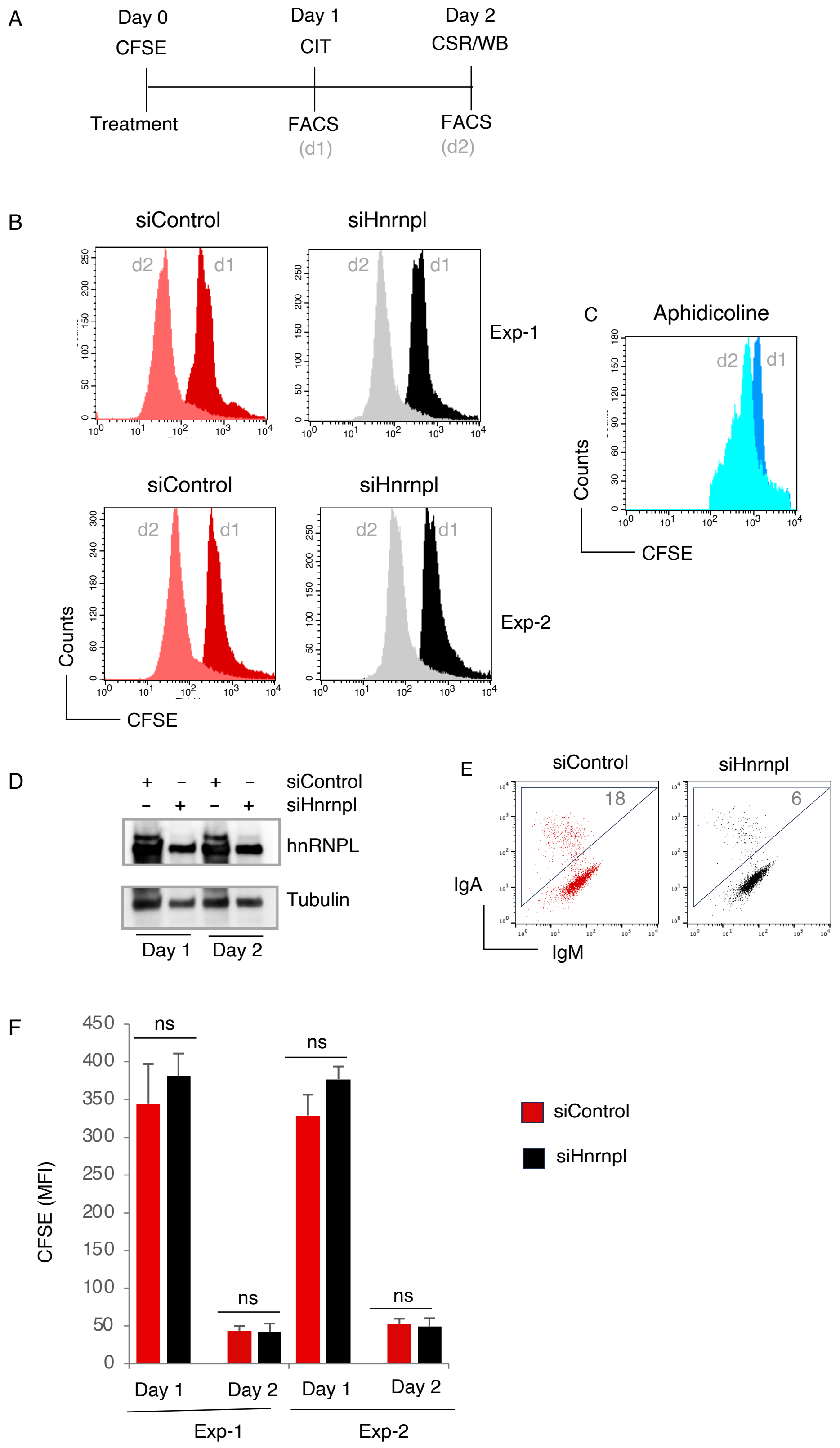

Supplementary Figure S8. Impact of hnRNPL knockdown on CH12F3-2A cell proliferation.

(A) Experimental timeline. **(B)** Representative FACS histograms from two independent experiments showing CFSE dye dilution in siControl- and siHnrnpl-treated CH12F3-2A cells. **(C)** CFSE-based proliferation assay of cells treated in parallel with the replication inhibitor aphidicolin. **(D)** Representative western blot showing hnRNPL knockdown efficiency relative to control. **(E)** FACS analysis confirming impaired CIT-induced IgM-to-IgA class switching upon hnRNPL knockdown under the same conditions. **(F)** Summary of CFSE assay data from two independent experiments. Bar graphs represent mean fluorescence intensity (MFI). Statistical analysis by unpaired two-tailed t-test ($n = 2$; mean ± SD; ns, not significant).

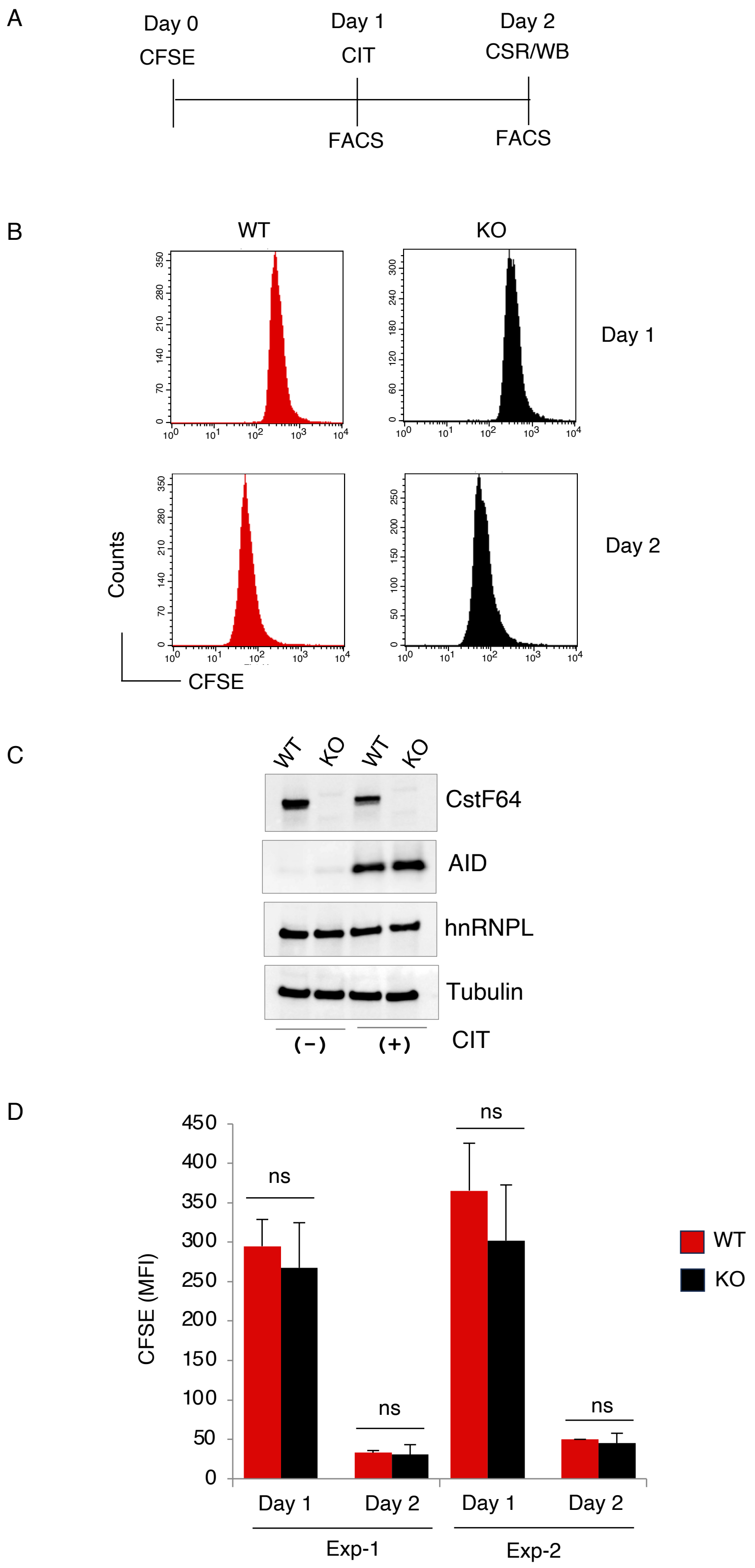

Supplementary Figure S9. Impact of CstF64 deficiency on CH12F3-2A cell proliferation.

(A) Experimental timeline. **(B)** Representative FACS histograms showing CFSE dye dilution in wild-type (WT) and *Cstf2* homozygous knockout (KO) CH12F3-2A cells. **(C)** Representative western blot confirming CstF64 loss and unaltered expression of AID and hnRNPL in the KO line. **(D)** Summary of CFSE assay data from two independent experiments. Bar graphs represent mean fluorescence intensity (MFI). Statistical analysis by unpaired two-tailed t-test ($n = 2$; mean $\pm$ SD; ns, not significant).

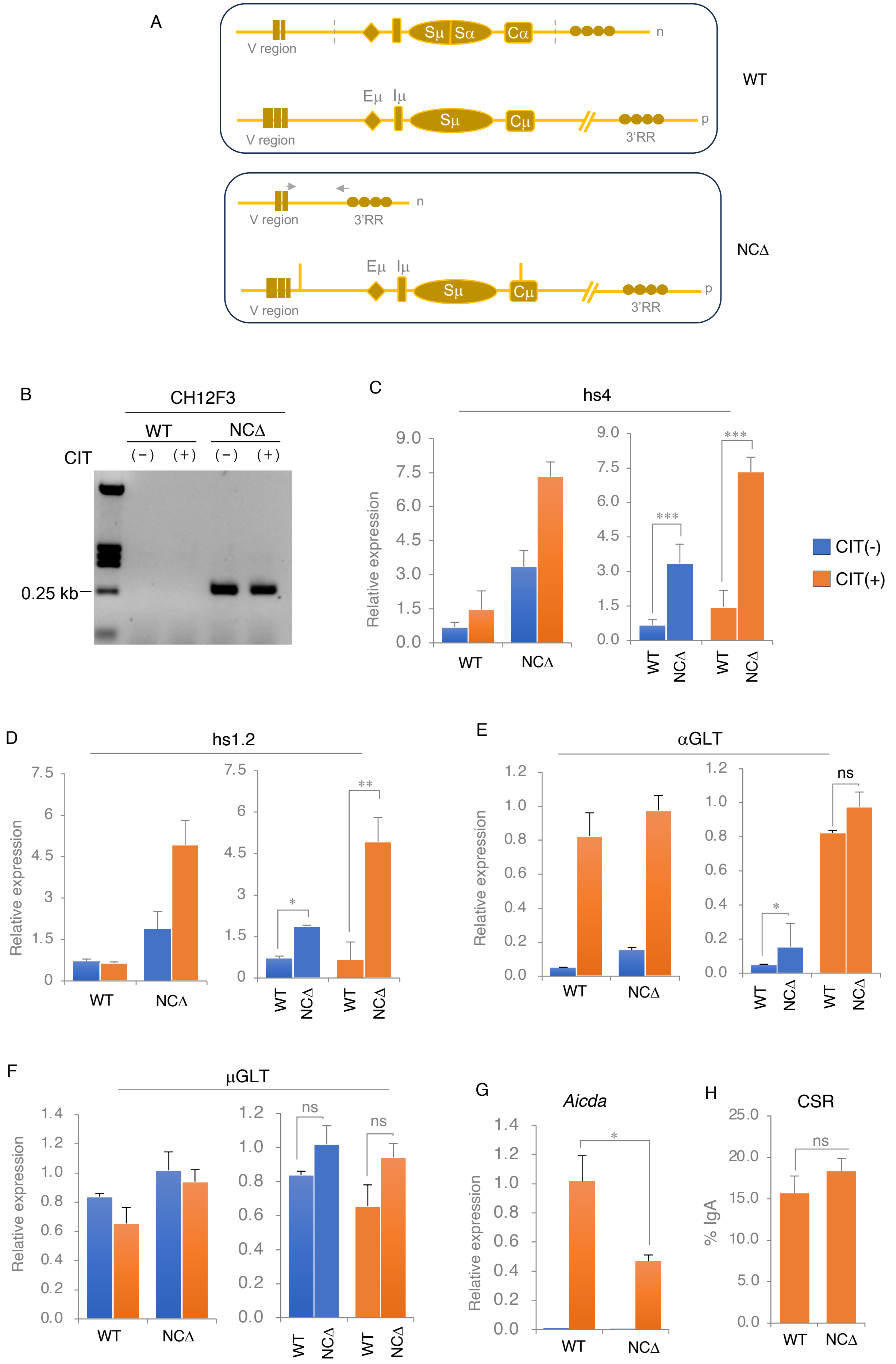

Supplementary Figure S10

Supplementary Figure S10. Comparison of CH12F3 NCΔ cells with CH12F3 WT control.

(A) Schematic of the *Igh* alleles in CH12F3 WT and CH12F3 NCΔ cells, adapted from Zhang *et al.* (2019, PMID: 31666703) and Dong *et al.* (2015, PMID: 26308889). Productive ("p") and non-productive ("n") alleles are indicated. In CH12F3 WT cells, Cas9-mediated deletion was targeted to the non-productive allele (dashed vertical lines) to generate the NCΔ line. Small arrows indicate the positions of the forward and reverse primers used for genotyping.

(B) Agarose gel showing PCR genotyping results using the primers shown in (A). A ~300 bp band, specific to CH12F3 NCΔ cells, confirms successful deletion and distinguishes NCΔ from WT cells.

(C–F) RT-qPCR analyses comparing transcript levels between CH12F3 WT and NCΔ cells under constitutive [CIT(–)] and inducible [CIT(+)] conditions.

(C) Expression of *hs4* enhancer RNA (hs4-eRNA). Constitutive expression is ~7 times higher in NCΔ cells, while CIT stimulation induces a ~2 times increase in both cell lines.

(D) Expression of *hs1.2* enhancer RNA (hs1.2-eRNA). Constitutive expression is higher in NCΔ cells, while CIT stimulation induces it further in NCΔ cell line.

(E) Expression of α germline transcripts (αGLTs). Slightly elevated in NCΔ cells under CIT(–), but strongly and comparably induced in both lines upon CIT stimulation.

(F) Expression of μ germline transcripts (μGLTs). No significant differences were observed between the two lines or across conditions.

(G) Expression of *Aicda* (AID). AID was robustly induced by CIT in both cell lines, though slightly lower in NCΔ. Under CIT(–) conditions, AID expression was minimal or undetectable in both lines, with bar plots nearly invisible.

(H) Flow cytometric analysis confirms comparable IgM-to-IgA class switch recombination (CSR) efficiency in CH12F3 WT and NCΔ cells, despite differences in AID expression.

All RT-qPCR data are normalized to the *Hprt* gene. Statistical analysis was performed using an unpaired two-tailed t-test (n = 3; mean ± SD; *$P \leq 0.05$; **$P \leq 0.01$; ***$P \leq 0.001$; ns, not significant).
